# Hydrogels for Three-Dimensional Ionizing-Radiation Dosimetry

**DOI:** 10.3390/gels7020074

**Published:** 2021-06-21

**Authors:** Maurizio Marrale, Francesco d’Errico

**Affiliations:** 1 Department of Physics and Chemistry, “Emilio Segrè” ATeN Center, University of Palermo, 90128 Palermo, Italy; 2 Istituto Nazionale di Fisica Nucleare (INFN), Sezione di Catania, 95123 Catania, Italy; 3 Scuola di Ingegneria, Università degli Studi di Pisa, 56126 Pisa, Italy; francesco.derrico@ing.unipi.it; 4 Istituto Nazionale di Fisica Nucleare (INFN), Sezione di Pisa, 56127 Pisa, Italy; 5 School of Medicine, Yale University New Haven, CT 06510, USA

**Keywords:** three-dimensional dosimetry, poly-vinyl alcohol, glutaraldehyde, ferrous sulfate, xylenol-orange, polyacrylamide gel, spectrophotometry, optical tomography, magnetic resonance imaging

## Abstract

Radiation-sensitive gels are among the most recent and promising developments for radiation therapy (RT) dosimetry. RT dosimetry has the twofold goal of ensuring the quality of the treatment and the radiation protection of the patient. Benchmark dosimetry for acceptance testing and commissioning of RT systems is still based on ionization chambers. However, even the smallest chambers cannot resolve the steep dose gradients of up to 30–50% per mm generated with the most advanced techniques. While a multitude of systems based, e.g., on luminescence, silicon diodes and radiochromic materials have been developed, they do not allow the truly continuous 3D dose measurements offered by radiation-sensitive gels. The gels are tissue equivalent, so they also serve as phantoms, and their response is largely independent of radiation quality and dose rate. Some of them are infused with ferrous sulfate and rely on the radiation-induced oxidation of ferrous ions to ferric ions (Fricke-gels). Other formulations consist of monomers dispersed in a gelatinous medium (Polyacrylamide gels) and rely on radiation-induced polymerization, which creates a stable polymer structure. In both gel types, irradiation causes changes in proton relaxation rates that are proportional to locally absorbed dose and can be imaged using magnetic resonance imaging (MRI). Changes in color and/or opacification of the gels also occur upon irradiation, allowing the use of optical tomography techniques. In this work, we review both Fricke and polyacrylamide gels with emphasis on their chemical and physical properties and on their applications for radiation dosimetry.

## 1. Introduction

Close to 6 million complete courses of radiation therapy are delivered every year worldwide. While this number represents less than 0.5% of the number of diagnostic procedures, it implies a major societal relevance since it means that ≃15% of the population undergoes radiation therapy during their lifetime in countries having Health Care Level I in the UNSCEAR global population model [1].

Radiotherapy involves a complex but well-established sequence of procedures comprising target identification, dose prescription, treatment planning, beam shaping and dose fractionation. The clinical success of radiotherapy and the protection of the patient are critically connected to delivering an optimal and uniform distribution of dose to the target volume while sparing the surrounding healthy tissues, particularly the most radiation-sensitive structures. Several studies demonstrate that morbidity is significantly related to the volume of irradiated normal tissue as well as to the actual dose delivered to the normal tissue. These findings have motivated research and development of techniques maximizing the irradiation of the target volume and minimizing the exposure of normal tissues. In the most modern radiotherapy techniques, such as intensity-modulated radiation therapy (IMRT), a variety of computer-controlled devices are used to produce multiple optimized, nonuniform and sometimes noncoplanar radiation beams that deliver conformal dose distributions around a target volume.

IMRT has encountered widespread acceptance and implementation both in the EU and in the US, promoted by evidence of superior tumor control and reduced toxicity, particularly in the treatment of head-and-neck and prostate cancers. However, this technology also poses some risks to the radiotherapy patient, deriving from a series of issues that have not yet been fully clarified [2].

One of the most prominent concerns is the lack of well-established 3-D imaging/ dosimetry techniques to verify the extremely complex conformal treatment plans. Of concern is also the radiation protection of patients. IMRT delivers a lower dose to normal tissues around the tumor target, compared with conventional radiation therapy, but it exposes patients to a higher whole-body irradiation. Thus, dosimetry in radiotherapy has the twofold goal of ensuring the quality of the treatment as well as the radiation protection of the patient. Dose measurements are crucial since treatment planning software is rarely accurate beyond some centimeters from the edge of the treatment volume.

Reference dosimetry for the acceptance testing and commissioning of IMRT systems is still based on ionization-chamber measurements, just like in conventional radiotherapy. However, particular care must be exercised when using ionization chambers in IMRT. Even the smallest chambers cannot resolve the steep dose gradients of up to 30–50% per mm generated with IMRT. To avoid volume-averaging effects, the chamber cross section must be smaller than the uniform dose regions in which it is placed. Moreover, the chambers are anisotropic and their irradiation with beams impinging from multiple directions requires an accurate knowledge of the angular dependence of the response [3].

Silicon diodes have also been used in IMRT, thanks to their appealing small size [4]. Like ionization chambers, diodes present the inherent constraint of being single-point dosemeters. This is not a limiting factor in conventional radiotherapy, where a significant portion of quality assurance dosimetry can be performed by scanning static beams in a water phantom. However, the limits of single-point measuring devices are approached when it comes to scanning the complex dose gradients generated by IMRT. This has prompted the development of a variety of novel linear-array and planar-array designs [5].

Next to ionization chambers, thermoluminescence dosimeters (TLDs) can measure integrated doses with accuracy and precision in the order of 3%, as required in clinical practice [6]. TLDs can be used for 3-D mapping of the dose distributions by placing large numbers of chips simultaneously in a phantom and then scanning them with automated readers. For these reasons, they have been widely used for the quantitative dosimetric verification of IMRT planning and delivery systems. However, using these detectors is an extremely laborious procedure and great care must be exercised in determining calibration factors for each individual detector chip.

Two-dimensional detectors can be used in the evaluation of the high-gradient regions of the IMRT dose distribution, which is crucial for quality assurance and cannot be done with point dosemeters like ionization chambers. In particular, radiochromic films present a fairly water-equivalent response, combined with high spatial resolution, direct development upon irradiation, insensitivity to visible light and linearity of response [7]. Owing to these properties, radiochromic films have been increasingly used in clinical dosimetry, including IMRT applications. Some concerns remain regarding the kinetics of signal development, whereby films irradiated to lower doses develop more rapidly than those receiving higher doses. In addition, the techniques required for accurate dose measurements with radiochromic films have not been universally adopted, although usage guidelines have been formulated.

A true 3D dosimeter may be identified via the “Resolution-Time-Accuracy-Precision (RTAP) criteria” [8], according to which, a three-dimensional dosimeter must be able to deliver “a 3D dosimetric analysis of a treatment plan with 1 mm isotropic spatial resolution, within 1 hour, with an accuracy of within 3% of the true value, with 1% precision”.

The radiation-sensitive gels described in this review fulfill the RTAP criteria and are considered the only true 3D dosimeters available nowadays for radiotherapy applications. These gels were introduced in the 1980s [9,10], they present tissue equivalent composition and density, so they also serve as phantoms, and their response is largely independent of radiation quality and dose rate. The original formulations are infused with ferrous sulfate and rely on the radiation-induced oxidation of ferrous ions to ferric ions (Fricke-gels) [9,11,12]. Later versions consist of monomers and cross-linkers dispersed in a gelatinous medium and rely on radiation-induced polymerization, which creates a stable polymer structure in response to free radicals generated by water radiolysis [13,14]. With both categories of gels, hereafter referred to as Fricke gels and Polyacrylamide gels (sometimes simply referred to as Polymer gels), irradiation causes variations of water-proton nuclear magnetic resonance relaxation times. These variations correlate with the dose absorbed locally and can be imaged using Magnetic Resonance Imaging (MRI). Changes in color and/or opacification of the gels also occur upon irradiation, allowing the use of optical tomography or spectrophotometry techniques.

## 2. Fricke Gels

### 2.1. Radiation Chemistry

The idea of a 3D dosimeter, a tissue equivalent material that is also sensitive to radiation throughout its volume, dates back to 1984 when Gore et al. [9] infused a hydrogel matrix with an acidic water solution of an iron salt [15]. Gore’s group demonstrated that the radiation-induced chemical changes in the gel could be imaged by nuclear magnetic resonance (NMR) techniques. Prior to the irradiation, the solution contains only Fe${}^{2+}$ ions that are oxidized to Fe${}^{3+}$ by the interaction of ionizing radiation with the dosimeter. The final concentration of Fe${}^{3+}$ ions is proportional to the absorbed dose of ionizing radiation. Radiations interact almost exclusively with the aqueous component of the hydrogels and thus create free radicals. Since water is the main constituent of biological soft tissues, Fricke-gels are almost perfectly tissue equivalent.

The free radicals (indicated with the symbol ${}^{•}$ after their chemical formula). produced by water radiolysis are highly reactive chemical species that eventually interact with the ferrous ions and oxidize them. When the solution is irradiated, water decomposes as follows:

(1) $$H_{2}O+h\nu \to H^{•}+{\mathrm{HO}}^{•}$$

Hydrogen atoms then react with oxygen to produce hydroperoxy radicals:

(2) $$H^{•}+O_{2}\to {\mathrm{HO}}_{2}^{•}\;$$

After these initial interactions between ionizing radiation and water, different chemical routes may lead to the oxidation of a ferrous ion. Some of them are:

(3) $${\mathrm{Fe}}^{2+}+{\mathrm{OH}}^{•}\;\to \;{\mathrm{Fe}}^{3+}+\;{\mathrm{OH}}^{-}\;$$

(4) $${\mathrm{Fe}}^{2+}+{\mathrm{HO}}_{2}^{•}\;\to \;{\mathrm{Fe}}^{3+}+\;{\mathrm{HO}}_{2}^{-}\;$$

(5) $${\mathrm{HO}}_{2}^{-}\;+H_{3}O^{+}\;\to \;H_{2}O_{2}+\;H_{2}O$$

(6) $${\mathrm{Fe}}^{2+}+H_{2}O_{2}\;\to \;{\mathrm{Fe}}^{3+}+{\mathrm{OH}}^{•}\;+\;{\mathrm{OH}}^{-}$$

Regardless of the chemical route, the final concentration of Fe${}^{3+}$ ions is proportional to the energy deposited by radiation in the dosimeter. The relationship that links the change in Fe${}^{3+}$ concentration to the dose is:

(7) $$\left[{\mathrm{Fe}}^{3+}\right]=\frac{10\;D\cdot G\left({\mathrm{Fe}}^{3+}\right)\cdot \rho }{N_{A}e}$$

where D is the dose, G (Fe${}^{3+}$) is the chemical yield of Fe${}^{3+}$ (expressed as ions produced per 100 eV), ρ is the density in kg/L, $N_{A}$ is Avogadro’s constant and *e* is the number of joules per electronvolt. The Fe${}^{3+}$ concentration in Fricke solutions is usually evaluated by spectrophotometric measurements in the UV range, since Fe${}^{3+}$ has absorption peaks at 304 nm and 224 nm. A gel, on the other hand, may be imaged by NMR to retrieve both the dose and its spatial distribution, since the magnetic properties of iron ions change with the oxidation state.

**Figure 1:**
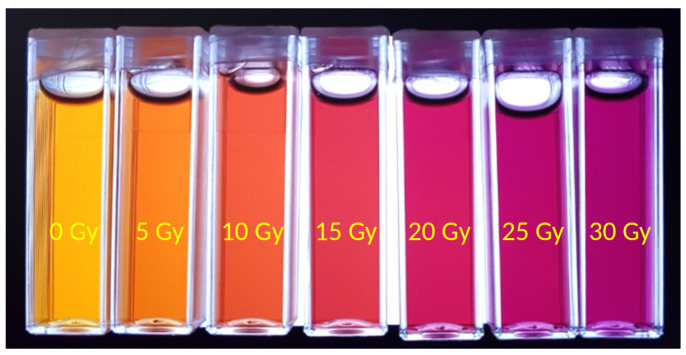
Photograph of Fricke gel samples irradiated up to 30 Gy (from left to right) at 5 Gy increments.

**Figure 2:**
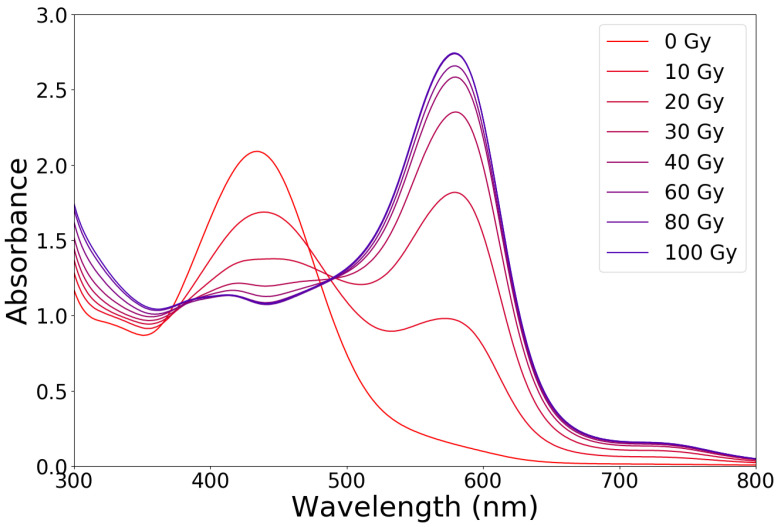
Typical UV-VIS absorption spectra of Fricke gels containing xylenol orange and irradiated to absorbed doses up to 100 Gy.

In the early versions of these dosimeters, the rapid diffusion of the Fe${}^{3+}$ ions inside the matrix restricted the timeframe for imaging to about 1 hour before the spatial information was unacceptably blurred. Various investigators proposed modifications to the standard formulation of Fricke solutions and gels by the addition of chelating agents [10,16], such as xylenol orange (XO), molecules that can form two or more coordination bonds with a metal ion. The chelated molecule is much larger than the Fe${}^{3+}$ ion alone; therefore, its mobility inside the polymer network is lower [16,17]. When added to the Fricke solution, XO chelates the Fe${}^{3+}$ ions creating a chemical species that has an intense absorption peak at 585 nm. Fricke solutions with the addition of XO can be infused in a gel matrix to obtain a compound that changes its color from yellow-orange to deep purple upon irradiation (Figure 1). Therefore, xylenol orange makes it possible to measure absorbed doses by means of UV-VIS spectrophotometry (Figure 2) and also to scan the gels with optical computed tomography (OCT) [18], a technique that offers lower imaging time compared to NMR [19] and comparable accuracy and spatial resolution.

### 2.2. Formulations

Historically, the most widely-used matrices for Fricke-infused gels have been two natural compounds: gelatine—a hydrolyzed form of collagen extracted from animal skin, bones, and connective tissues, and agarose—a polysaccharide polymer extracted from seaweed.

The natural origin of gelatine involves an inherent variability in purity and composition due to age and species of the animal source and due to the complexity of extraction and purification processes. Different gelatine batches present slightly different natures, and therefore gelatine-based products inevitably present variable characteristics. Spontaneous gelling of gelatine involves the creation of interchain links, mainly hydrogen bonds, between the functional groups of the amino-acid residues of the chains. Since gelatine extraction processes yield variable length chains, crosslinking, diffusion and permeability characteristics are all affected. External cross-linking agents may also be used, but cross-linking is problematic because of the chemical and structural complexity of gelatine. A further drawback encountered with gelatine formulations is their degradation over time.

Agarose has also been widely used as a matrix for Fricke-infused gels; indeed, some agarose gels are reportedly more sensitive to radiation than either aqueous solutions or gelatine systems infused with ferrous sulfate [20]. However, agarose also presents a series of drawbacks: first of all, as with gelatine, its natural origin affects the reproducibility of its structure and of its cross-linking. Moreover, agarose gels are translucent, rather than transparent, which hinders optical absorbance measurements [21]. A further drawback is that agarose must be raised to high temperatures (90–95 °C) to properly dissolve and then form a gel while cooling down, which may cause a loss of dissolved oxygen [22].

Because of the above-listed limitations of gelatine and agarose, recent research has focused on synthetic matrices and has aimed at developing dosimetric gel formulations based on poly-vinyl alcohol (PVA), offering high stability, sensitivity and ease of manufacture. PVA is a polymer with a simple chemical structure, it is water-soluble, nontoxic, inexpensive and suitable for manufacturing hydrogels via physical or chemical routes. PVA is synthesized through well-established processes that allow an accurate selection of the molecular weight distribution, i.e., the length of the chains. Consistency between different batches is very high and so is the reproducibility of derived products.

The easiest physical method to produce PVA gels is subjecting an aqueous solution of PVA to freeze-thaw cycles. At low temperature, segments of PVA chains are coordinated into microcrystalline structures that act as cross-links. The final characteristics of these hydrogels depend on various parameters, such as the number of thermal cycles, the temperature reached in the cycles, the cooling and heating rates and the concentration of the polymer and its molecular weight.

Alternatively, the production of PVA hydrogels may be achieved by chemical pathways, using suitable cross-linking agents. Among them is glutaraldehyde (GTA), a small molecule that easily reacts with the PVA hydroxyl groups, creating bridges between the chains [23]. GTA is a relatively nontoxic substance and its PVA cross-linking reaction occurs at room temperature, yielding hydrogels that are transparent to light. Several characteristics of the GTA-PVA gels depend on the degree of cross-linking and can be easily modulated by adjusting the concentration of PVA, its molecular weight and the GTA/PVA ratio.

### 2.3. Characterization and Performance

The dosimetric gel properties described hereafter are mainly based on dosimeters made with polyvinyl-alcohol cross-linked by adding glutaraldehyde (GTA) [24,25] and infused with a Fricke-solution (25 mM sulfuric acid and 0.5 mM iron ammonium sulfate), along with 0.165 mM xylenol-orange (XO).

#### 2.3.1. Gelation Process

The above-mentioned PVA-GTA formulation is liquid when kept refrigerated and undergoes gelation when raised to room temperature. The gelation process of these gels is strongly affected by two main parameters, temperature and GTA concentration.

Table 1 reports the gelation times of gels produced with different concentrations of GTA and kept at different gelation temperatures [26]. The viscosity of the PVA-GTA solution starts to increase right after the addition of GTA and ramps up when the solution approaches the gelation point; therefore, the time effectively available to pour the PVA-GTA solution into a container is shorter than the nominal gelation time. Solutions with ≤26.500 mM of GTA do not turn into a gel if they are kept at temperatures ≤15.0 °C. This allows pouring them into containers (phantoms) with large volumes or complex shapes: reagents are mixed keeping the solution below 15 °C, then the temperature is slowly raised after casting.

#### 2.3.2. Reagent Purity and Reproducibility

While the use of synthetic reagents allows for the production of reproducible gels, great care must be exercised in the selection and storage of the reagents. The use of fresh, analytical-grade-purity chemicals is in general required.

Aging effects are particularly noticeable in the case of glutaraldehyde and they affect the absorbance level of nonirradiated gels. GTA is known to undergo self-polymerization, a process that is accelerated by inadequate storage [27]. Self-polymerization of GTA is significantly slower in the absence of oxygen and storage under an inert gas is an effective method to extend the shelf life of GTA. A further option is freezing [27,28,29]; this way, the original properties of GTA may be preserved for up to four months even with multiple freezing-thawing cycles.

Poly-vinyl alcohol is synthesized through well-established processes that allow an accurate selection of the molecular weight distribution, i.e., the length of the chains, in turn allowing a high-consistency between different batches and a high reproducibility of derived products. However, even high-purity-grade PVA may contain some contaminant residues: these impurities are reported all together as “residue on ignition”, and the most abundant is sodium acetate, since the majority of the ashes are sodium oxide [30,31,32].

Higher-purity commercial versions of poly(vinyl alcohol) can be found, such as that denominated Mowiol (Sigma Aldrich), whose ignition residue is ≤0.5%. This higher-purity PVA offers a variety of advantages [33], including a lower initial absorbance at zero-dose, the production of higher-concentration gels without rapid oxidation effects, an easier dissolution at atmospheric pressure without the need for an autoclave, a lower viscosity and easier pourability of the resulting solution in the containers of interest, the availability in wide range of molecular weights and also a lower cost compared to standard PVA.

As is the case with glutaraldehyde and poly(vinyl alcohol), xylenol orange must be chosen carefully in the production of dosimetric gels. Indeed, the chemical purity xylenol orange was found to vary significantly not only between manufacturers but also from batch to batch for the same product [34].

#### 2.3.3. Response to Ionizing Radiation

**Figure 3:**
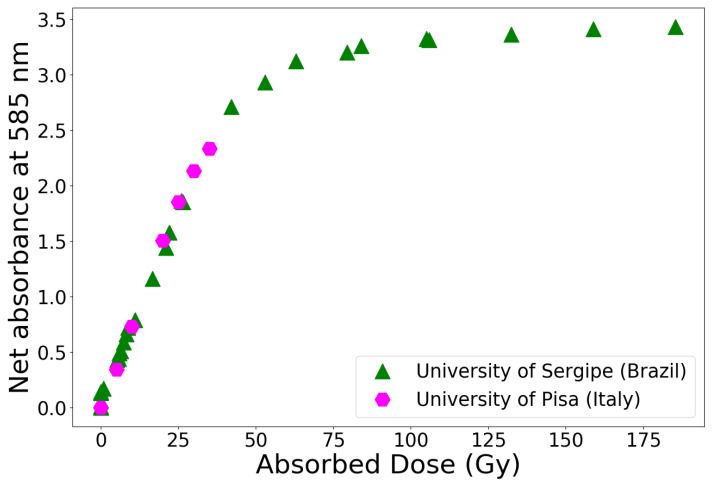
Dose response of PVA- GTA Fricke gels measured with UV-VIS spectrometry at the University of Pisa, Italy, and at the Federal University of Sergipe, Brazil; onset of saturation effects may be observed around 30 Gy. Error bars (1 standard deviation) are included in the plot symbols. Adapted from [24].

The response PVA-GTA Fricke gels (Figure 3) is quite similar to that of formulations based on gelatine; however, PVA-GTA gels present a significantly lower diffusion of ferric ions post-irradiation and a high reproducibility of the sensitivity between different dosimeter batches [26].

Since the amount of inorganic reagents in the gels is negligible in relation to the overall composition, ionizing radiations interact almost exclusively with the aqueous medium. Thus, Fricke gels in general, and PVA-GTA gels in particular, are almost perfectly tissue equivalent [26,35,36] and this contributes to their excellent dosimetric performance [24,37].

The minimum detectable dose (MDD) can be evaluated for each gel batch as MDD=3σ/s, where σ is the standard deviation of the absorbance measurements of unirradiated samples and s is the slope of the linear regression of the dose-response curve of each batch. Reported values of the minimum detectable dose are on average 0.122±0.003 Gy. This MDD, combined with an upper limit $D_{max}=30$ Gy for the linear region of the response, corresponds to a dynamic range $D_{max}/MDD>200$. The latter is adequate to map 3D dose distributions in radiotherapy, including the tumor target region that must be irradiated and the surrounding healthy tissue regions that must be spared.

Even better results in terms of dose response are achieved when using PVA of the Mowiol type [25,26], rather than standard PVA. The dose-response of 10% *w*/*v* and 12.5% *w*/*v* Mowiol-based gels is linear up to 25 Gy (R${}^{2}$ > 0.99), with a sensitivity of 0.078±0.001 Gy${}^{-1}$ and 0.077±0.001 Gy${}^{-1}$, respectively. These values are slightly higher than 0.074±0.001 Gy${}^{-1}$, i.e., the average value found for 10% *w*/*v* PVA gels. This excellent dosimetric performance of Mowiol-PVA gels was also independently verified by another group adopting the same formulation [36].

#### 2.3.4. Ferric Ion Diffusion Effects

Fricke gel formulations suffer from the diffusion of ferric ions after irradiation [38]. This effect is contrasted by the use of chelating agents such as xylenol-orange. A variety of experimental methods have been described to derive the ion diffusion coefficients from dose maps measured either by magnetic resonance or optical techniques, including analytical approximations to the theoretical distribution, Bayesian estimations and finite element methods [39,40,41,42,43,44].

**Figure 4:**
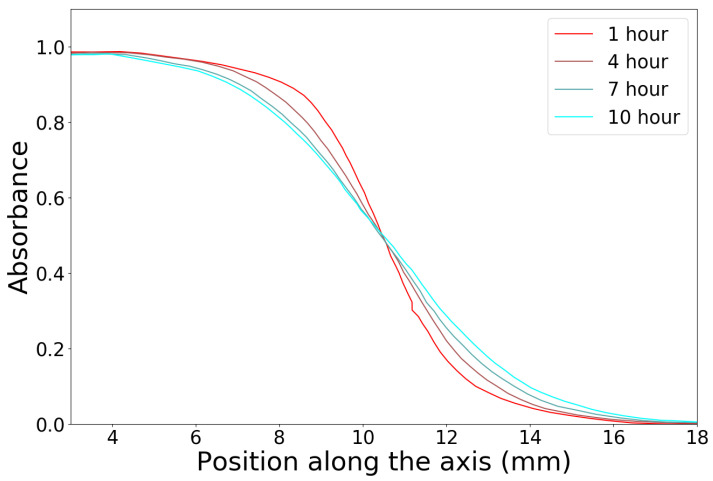
Normalized time-dependent absorbance-position profiles for PVA-B gels. Adapted from [26].

In a widely used approach, a high dose of high-energy X-rays is delivered to partially shielded cuvettes. A portion of the cuvettes is covered with lead, while the rest is left unshielded. An example of time-dependent absorbance profiles is shown in Figure 4 for PVA-B gels. After Kron et al. [40], the profiles are fitted with an inverse square root function of the form:

(8) $$A=A_{min}+\frac{1}{2}\left(A_{max}-A_{min}\right)\left[1+\frac{x-x_{0}}{\sqrt{{\left(x-x_{0}\right)}^{2}+n}}\right]$$

where $A_{min}$ and $A_{max}$ are the minimum and maximum measured absorbance values, $x_{0}$ is the lateral shifting of the inflection point, and *n* is the “curvature parameter”. The latter varies linearly with time, and this variation relates directly to the diffusion coefficient. Namely, the diffusion coefficient of the gel is equal to the rate of change of *n* multiplied by 0.212.

Results reported in Table 2 show that the concentration of GTA affects the degree of crosslinking of the matrix and thus the diffusion coefficient of the gel. Hence, controlling the cross-linking degree by means of the GTA concentration permits a reduction of the diffusion coefficient: as the crosslinking degree increases, so does the number of obstacles that the Fe${}^{3+}$-xylenol-orange complex may encounter. This increased crosslinking density slows down the movement of the complex because the probability to find an obstacle instead of a free path is higher in a gel with a higher crosslinking degree.

Table 2 also shows that the diffusion coefficient decreases while the molecular weight of PVA increases. The difference may be due to two phenomena. Longer chains create a more interconnected network; therefore, the diffusing species has a higher probability to encounter the polymer chain instead of a free path inside the solvent while diffusing. Furthermore, the length of the chains affects their random coil configuration. The probability that the GTA will link two different PVA chains, instead of linking two extremities of the same chain, may change with different random coil configurations.

As indicated in Table 2, Fricke gels produced with the PVA-A formulation and examined with spectrophotometry yielded a diffusion coefficient of 0.171±0.012 mm${}^{2}$ h${}^{-1}$. When gels produced with the same PVA-A formulation are similarly irradiated and then scanned with a 7 T MRI scanner, the outcome is a diffusion coefficient of 0.18 mm${}^{2}$ h${}^{-1}$ [45]. Achieving virtually the same result through completely independent methods demonstrates the reliability and reproducibility of this gel matrix.

The diffusion coefficients of Mw-A and Mw-B gels were 0.184±0.014 mm${}^{2}$ h${}^{-1}$ and 0.152±0.008 mm${}^{2}$ h${}^{-1}$, respectively (in order to keep the crosslinking degree constant, the GTA concentration was increased to 33.125 mM in the Mw-B formulation).

#### 2.3.5. Spontaneous Oxidation

In addition to the diffusion of ferric ions, Fricke gels suffer from spontaneous oxidation. The conversion from Fe${}^{2+}$ to Fe${}^{3+}$ occurs spontaneously even in the absence of irradiation, because the ferric state is the more stable one.

**Figure 5:**
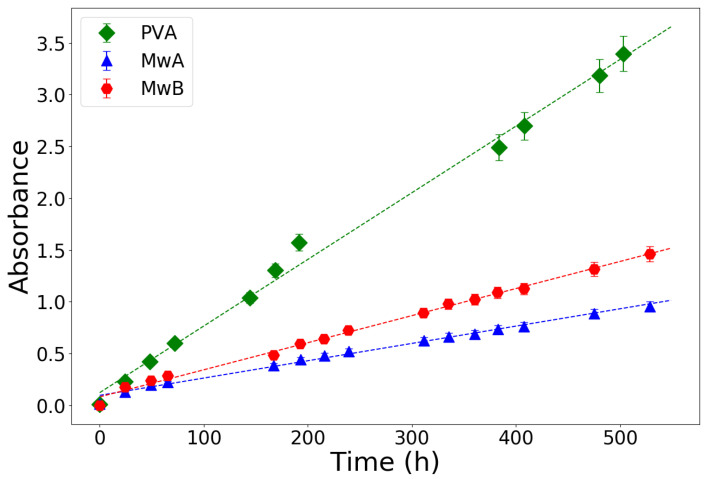
Spontaneous oxidation rate of various PVA and Mowiol gels. Adapted from [26].

Figure 5 shows the spontaneous oxidation rate of PVA-A, Mw-A and Mw-B gels. The spontaneous oxidation rates of Mw-A and Mw-B were $1.66\times 10^{-4}$ h${}^{-1}$ and 2.60 h${}^{-1}$, respectively. These values are about 4 and 2.5 times lower than the oxidation rate of PVA-A ($6.40\times 10^{-4}$ h${}^{-1}$). Moreover, the oxidation rate of gels made with Mowiol is over an order of magnitude lower than gels made with natural polymer, that have a typical oxidation rate of ∼$2\times 10^{-3}$ h${}^{-1}$ [46,47]. While the shelf-life of the Mowiol gels is not adequate for commercialization, yet, such a low oxidation rate allows producing gels and storing them for several weeks before usage.

A comparison of previously-described Fricke gels is presented in Table 3. When compared to gelatine gels, Mowiol-gels offer a considerably lower diffusion coefficient and oxidation rate without a reduction in sensitivity. Compared to standard PVA, Mowiol is easier to process and more suitable for the production of homogeneous large volume phantoms, while the higher sensitivity improves its dosimetric effectiveness.

In particular, Mw-B gels present a diffusion coefficient of 0.152±0.008 mm${}^{2}$ h${}^{-1}$, a sensitivity equal to 0.077±0.001 Gy${}^{-1}$ and a spontaneous oxidation of $2.60\times 10^{-4}$ h${}^{-1}$, offering arguably the best all-around performance of current Fricke-infused gels.

#### 2.3.6. Recent Developments: Functionalized, Doped and Reusable Fricke Gels

While research in recent years has mainly focused on polyacrilamide gel formulations, described in the next section, R&D on Fricke gels has continued nonetheless. In particular, a modification to the PVA hydrogel has been described [48] whereby the chelating agent xylenol orange is partially bonded to poly-vinyl alcohol. This functionalized poly-vinyl alcohol hydrogel presents a relatively low sensitivity of 0.014 Gy${}^{-1}$ but it offers a diffusion coefficient of 0.133 mm${}^{2}$ h${}^{-1}$, which adds approximately 1 hour to the useful timeframe between irradiation and readout. Another promising approach to the functionalization of the gel matrix was recently described by Lazzaroni et al. [50]. Alternatives to the use of xylenol orange are also actively investigated [51] and among them methylthymol blue appears to be a promising option [52].

In another development, nanocomposite Fricke gels have been prepared with different concentrations of nano-clay, perchloric acid and ferrous ions under deaerated conditions [53]. The radiological properties of these gels were examined by irradiations with carbon and argon ion beams spanning a linear-energy-transfer (LET) range of 10 to 3000 keV/µm. Contrary to conventional Fricke gel, the nanoparticle doped gels were found to present a response independent of the LET and nearly constant even at very high LET (3000 keV/µm) values in the Bragg-peak region of the argon ion beam.

The previously described formulations of PVA-GTA gels were the basis for the development of reusable Fricke gels aimed at reducing the time, effort and cost of manufacturing the dosimeters whenever they are required. Reusable gels are also appealing because they would permit the calibration of each individual dosimeter, rather than calibrating samples drawn from a batch of single-use gels.

A notable development in this respect is PVA-GTA gels added with a tri-iodide complex and glucono-δ-lactone acid [54]. The unirradiated gel samples are clear and transparent, while irradiated samples turn to a brownish hue with a peak absorbance response at 490 nm. The sensitivity of these gels is about 1/3 that of the previously described Mowiol-based compositions, i.e., s=0.025±0.001 Gy${}^{-1}$ and MDD=0.545±0.001 Gy. However, these gels can be annealed to almost complete transparency by thermal treatment at 45 °C for 24 h. These gels clearly require further investigations in order to explain, among other issues, the signal development kinetics. It is in fact observed that the absorbance decreases within one day post-irradiation and then gradually increases afterwards. The initial decrease is attributed to a reduction of poly-iodide ions to monoiodide ions mediated by GTA or fructose. Conversely, the ensuing increase in absorbance is attributed to either the oxidation of the iodide ion or reactions with light. Despite these open questions, the reversible formulations hold great promise and further studies are certainly warranted.

## 3. Polyacrylamide Gels

Polyacrylamide gels exploit the radiation-induced polymerization of monomers and crosslinkers dispersed in aqueous solutions. The first application of polymer systems in radiation measurements was performed in the 1950s when polymethylmethacrylate was exposed to ionizing radiations. First uses of nuclear magnetic resonance for detecting ionizing radiation exposure date back to 1992 when it was observed that the transverse relaxation times of an aqueous solution of N,N-methylene-bis-acrylamide (Bis) and agarose decrease with increasing absorbed dose. Successively, a different system composed of Bis, acrylamide (AAm), nitrous oxide and agarose was proposed as a gel dosimeter named BANANA. This system does not suffer from diffusion problems (as observed in Fricke gel dosimeters) and maintains the information on spatial dose distribution. An evolution of this BANANA gel dosimeter was realized by replacing agarose with gelatin and this gel was commercially named BANG. The noncommercial version of this polymer gel is called PAG [55].

Among the main issues influencing the accuracy of polyacrylamide gels are oxygen inhibition effects and MRI imaging artifacts [56]. Indeed, the response and sensitivity of polyacrylamide gel dosimeters is heavily affected by oxygen levels which can inhibit the polymerization processes. This is the reason why these polymeric gels must be prepared in an oxygen-free environment, for example, in a glove box flushed with inert gas such as nitrogen or argon [56].

A further step forward in the polyacrylamide gels production was achieved with the so-called normoxic gels (the first type was named MAGIC gel) in which a metallo-organic complex is used to bind the atmospheric oxygen [14]. This choice eliminates the issue of oxygen inhibition and allows for the preparation of gels on the benchtop in the laboratory. The MAGIC gel contains ascorbic acid (also known as vitamin C) which is able to bind free oxygen contained within the aqueous gelatin matrix giving rise to metallo-organic complexes also thanks to copper sulfate. Various other antioxidants were used to produce normoxic gels [55]. In the following sections, radiation chemistry, preparation procedure as well as dosimetric features of these formulations are examined.

### 3.1. Radiation Chemistry

Since gel dosimeters are mainly composed of water (usually about 90% by weight), an important contribution to the effects on the solutes derives from the indirect action of some radiation-induced species produced in water. Indeed, irradiation causes radiolysis, the breaking of water molecules, yielding highly-reactive radicals and ions [57]. Production of radiolytic species (such as excited water molecules (H${}_{2}$O${}^{*}$), ionized water molecules (H${}_{2}$O${}^{+}$) and subexcitation electrons, e${}^{-}$) occur on a timescale of femtoseconds and these products as well as their spatial distribution depend on the type of irradiation and the energy of the primary particles. The clusters of radicals observed after irradiation with X-rays, gamma rays and electrons have an average radius of 1 nm ( for these radiations, the energy released in each cluster ranges between 6 and 100 eV) and are termed “spurs”. Afterwards, the de-excitation of water molecules produces dissociation into H${}^{•}$ and OH${}^{•}$ radicals and possible production of hydrogen (H${}_{2}$) gas. These early-stage radiolytic products appear within 1 nm from the path of the incident ionizing particle. The successive stage, called chemical stage, is characterized by interactions among the various radiolytic products and their Brownian diffusion in the solution.

The average distance between free radicals increases with time. However, after $10^{-11}$ s reactive products achieve a local thermal equilibrium and recombination processes occur. In this time interval, the root mean square displacement of the particles is about 0.28 nm (based on an average diffusion coefficient of $4\times 10^{9}$ m${}^{2}$ s${}^{-1}$ for the reactive particles) which is about one order of magnitude smaller than the intermolecular distance of monomers in a polyacrylamide gel dosimeter [55,58]. After $10^{-8}$ s, the prevalent species are hydrated electrons (e${}_{aq}^{-}$), hydroxyl radicals (OH${}^{•}$) and hydrogen radicals (H${}^{•}$). Assuming that the diffusion coefficient for the radiolytic species in water is similar to that in the gel matrix [55,58], the displacement from the point of creation after $10^{-8}$ s is about 9 nm and consequently radicals react with the monomers.

Therefore, the dissociation into reactive species induced by irradiation (characterized by a dissociation rate, $k_{D}$, proportional to the absorbed dose) can be written as:

(9) $$H_{2}O\overset{k_{D}}{\to }2R^{•}$$

where R${}^{•}$ are the primary radicals e${}_{aq}^{-}$, OH${}^{•}$ and H${}^{•}$.

The primary radicals R${}^{•}$ are produced with a rate described by the following equation:

(10) $$R_{D}\left(R^{•}\right)=\left(G_{e-}+G_{\mathrm{OH}•}+G_{H•}\right)\frac{\mathrm{dD}}{\mathrm{dt}}m_{w}$$

where G is the chemical yield (number of particles per 100 eV of absorbed energy) of primary radicals ($e_{aq}^{-}$, OH${}^{•}$ and H${}^{•}$), $\frac{\mathrm{dD}}{\mathrm{dt}}$ is the dose rate and $m_{w}$ is the mass of the irradiated water.

The radical species induce the polymerization of monomers by reacting with the monomer. The first step can be written as follows:

(11) $$R^{•}+M\overset{k_{I}}{\to }M_{1}^{•}$$

characterized by a initiation reaction rate constant $k_{I}$. The primary radicals can also give rise to the polymerization of polymers containing a double bond by binding with an electron of the double bond:

(12) $$R^{•}+M_{n}\overset{k_{I}\left(n\right)}{\to }{\mathrm{RM}}_{n}^{•}$$

Polymers continue growing because the successive interactions of polymeric radicals add monomers or pendant vinyl groups (resulting from the crosslinker) that are present on other polymer chains.

The polymerization process terminates when two radicals react bringing about combination or disproportionation according to this reaction

(13) $$R^{•}+M_{n}^{•}\overset{k_{T}c\left(o,m\right)}{\to }M_{m}$$

If one vinyl group of the crosslinker molecules (such as Bis) is involved in polymerization reaction, the other one (which now is a pendant vinyl group along the polymer chain) becomes available for successive propagation reactions forming crosslinks. Sometimes, the second vinyl group can undergo a cyclization reaction after the polymerization of the first vinyl group and this reduces the number of pendant vinyl groups available for crosslinking.

The polymerization processes are heavily affected by the presence of oxygen in the gel. Indeed, in this case peroxide radicals are created:

(14) $$R^{•}+O_{2}\overset{k_{01}}{\to }{\mathrm{ROO}}^{•}$$

(15) $$M_{n}^{•}+O_{2}\overset{k_{02}}{\to }M_{n}{\mathrm{OO}}^{•}$$

and these will quickly react with other radicals rapidly bringing about the end of the polymerization process.

The presence of oxygen produces an inhibition in the MR dose response in the low dose region of polyacrylamide gel dosimeters. Various methods were adopted to eliminate oxygen such as preparation of gel solution in a inert gas (such as nitrogen or argon gas) atmosphere [13] or by using antioxidants[14]. More details about radiation chemistry in polyacrylamide gels can be found in literature [55,58,59,60,61].

### 3.2. Preparation

Polyacrylamide gel dosimeters are produced by dispersing monomers in a hydrogel matrix. Two aqueous solution aliquots of equal volume are prepared: one for dissolving gelatin and the other for the monomers. The crosslinker (such as Bis) is added to the monomer portion after heating it to 45 °C to favor crosslinker dissolution. The gelatin portion is also heated to 45 °C. For anoxic polyacrylamide gels, an inert gas such as nitrogen or argon is used to remove oxygen from both solutions and the residual oxygen level is checked to be below a target threshold (0.01 mg L${}^{-1}$). Afterwards, both solutions are cooled down to 32 °C and then mixed and poured into the phantom recipients inside a glove box. The latter is purged with nitrogen to avoid entrance of fresh oxygen inside the gel phantoms during filling. In the case of normoxic polyacrylamide gels, an antioxidant is added to the mixture while stirring and cooling down to 32 °C. In order to control the cooling process, the phantoms are immersed in a large container of water kept at 32 °C. Clearly, the phantom material should avoid oxygen infiltration and therefore it is usually glass or Barex (a thermoforming plastic with low oxygen permeability) [58].

### 3.3. Characterization and Performance

The main application field of polyacrylamide gels is clinical radiation therapy, which requires reliable dosimetry, adequate precision, high-resolution maps of 3D dose distribution, and verification of the computer-generated treatment plans for tumor irradiations [62,63]. While this review focuses on literature from the past decade, earlier studies are thoroughly examined by Baldock et al. [55].

**Figure 6:**
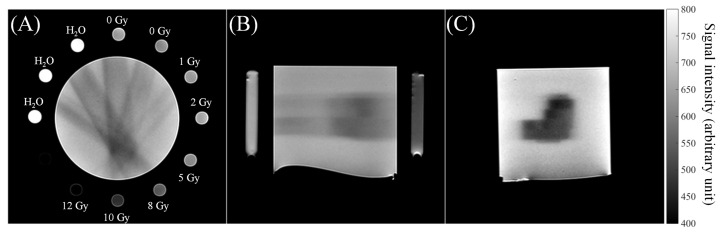
Examples of T2-weighted images of the irradiated NIPAM gel phantom: (**A**) Axial, (**B**) sagittal and (**C**) coronal images. The calibration tubes filled with NIPAM gel exposed to given doses (reported in Figure **A**) are located around the centran phantom. Reproduced from [64].

The polymerization process induced by ionizing radiation involves a reduction of the T${}_{2}$ relaxation time (and a related increase of the relaxation rate R${}_{2}$ = 1/T${}_{2}$) detailed in the following section “Magnetic Resonance Imaging”. This produces a hypointense signal in the regions exposed to radiation as can be seen in Figure 6.

**Figure 7:**
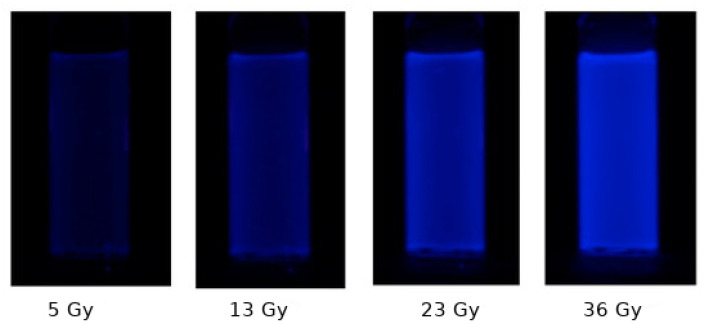
Examples of color fluorescence in UV light of maleimido-pyrene in tertiary-butyl acrylate, as a function of γ-photon dose. Adapted from [65].

Changes of optical and luminescence properties can also be observed in irradiated polymer-gels. Figure 7 shows how the fluorescence induced by UV light in maleimido-pyrene in tertiary-butyl acrylate gels changes with dose in γ-irradiated gels.

In order to quantify the response accuracy, the “gamma index” defined by Low et al. [66] is often used. The latter includes both spatial and dosimetric features of the detector and compares its response to the spatial dose distribution produced by a treatment planning system. Quantitative assessments based on dosimetric gels may be affected by factors that induce deviations between prescribed dose and measured dose (such as chemical stability, spatial stability, energy-dependent response, dose rate-dependent response, temperature dependency, etc.) and by factors leading to spatial deviations (such as phantom positioning error, volumetric contraction of the gel dosimeter and imaging artifacts).

For the characterization of the response to ionizing radiations, two quantities are usually adopted: the dose resolution and the sensitivity. The dose resolution is the minimum separation of two dose levels detectable with a certain level of confidence, p, and represents the ability to distinguish two similar but distinct dose values. Different values have been reported for different gels types and different readout techniques [67,68,69,70]. Among the best values, are detectable differences of 0.2–0.3 Gy over a 0–30 Gy dose range obtained for a 2-propanol-based gel dosimeter formulation (N-isopropylacrylamide, NIPAM, monomer) [68] and 0.085 and 0.190 Gy, for 30 Gy and 45 Gy, respectively, for a polymer gel dosimeter with cross-linked poly-vinyl alcohol [71].

The sensitivity of polyacrylamide gels is defined as the slope of the linear region of the dose response curve [55]. This feature also depends on the type of gel and on the experimental technique used to measure the dose. In the case of MRI acquisitions, the sensitivity is the slope of the R${}_{2}$ dose response curve and is reported as Gy${}^{-1}$ s${}^{-1}$ and was found to be in a range between 0.03 and 8.93 Gy${}^{-1}$ s${}^{-1}$ [55,62,72,73,74,75,76,77,78,79]. Recently, addition of high atomic elements (such as bismuth, gadolinium, silver or gold nanoparticles, etc.) has been investigated for increasing photon sensitivity [80,81,82,83,84,85,86,87,88,89,90,91,92].

**Figure 8:**
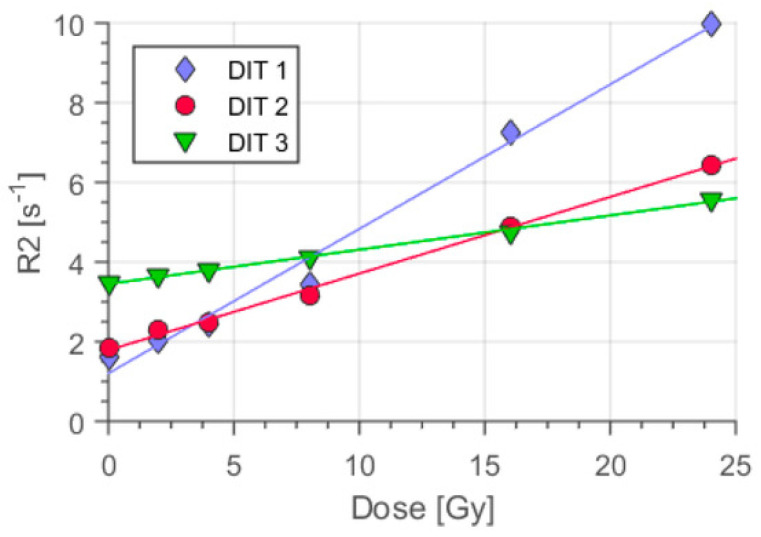
Dosimetric response of methacrylic acid gels produced with different concentrations of dithiothreitol oxygen scavenger (DIT1: 2 mmol/kg, DIT2: 10 mmol/kg, and DIT3: 50 mmol/kg). Reproduced from [93].

An example of the response curve obtained through MRI of a polyacrylamide gel (Dithiothreitol and Methacrylic Acid, MAGADIT) exposed to linear accelerator (LINAC) photons is shown in Figure 8 [93]. The effect of the Dithiothreitol oxygen scavenger was also investigated: the sensitivity reduction seen in Figure 8 with increasing dithio concentration can be attributed to interactions of this oxygen scavengers with radicals (with following reduction of the number of polymerization initiating radicals).

**Figure 9:**
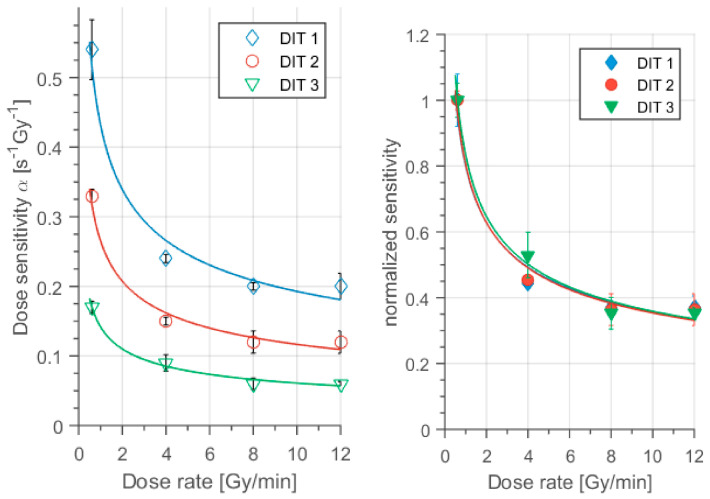
Dependence of the sensitivity of Dithiothreitol and Methacrylic Acid (MAGADIT) on the dose rate. Reproduced from [93].

Since different regions of the gels are exposed to different doses and dose rates, the response of polyacrylamide gels for 3D dose determinations should be independent of dose rate. A dependence on the dose rate would lead to inaccurate dose maps. Some gels such as BANG-2, NIPAM, PAGAT (PolyAcrylamide Gelatin gel fabricated at ATmospheric conditions) and PASSAG (Poly 2-Acrylamido-2-MethylPropane Sulfonic acid-Sodium Salt and Gelatin) gels show response independent of dose rate, whereas some others such as MAGAT (which comprises methacrylic acid, hydroquinone, gelatin and tetrakis hydroxymethyl phosponium chloride, THPC) have a dose rate dependent response and should be used only for doses below 2 Gy [55,76,94,95,96,97] or for dose rates above 4 Gy/min as MAGADIT [93]. An example of the dependence of MR sensitivity on dose rate is shown in Figure 9.

For many polyacrylamide gels, the response to photons and electron is independent of the energy of the beams [55,98,99,100]. Other polymer gels do show a dependence on beam energy, among these are the nMAG (normoxic gels based on methacrylic acid) and MAGIC-A (MAGIC and agarose) formulations [55]. Other factors to be evaluated are the intrabatch and the interbatch reproducibilities. These were found to be of the order of few percents (in many cases well below 5%) [55,101,102].

The use of polyacrylamide gel dosimeters in routine clinical applications is not widespread because of the toxicity of the required monomers [94]. Monomers such as acrylamide and Bis are toxic (acrylamide is a serious neurotoxin and a suspected human teratogen and carcinogen) and therefore gels containing them should be prepared, used and handled with great care. Major efforts are devoted to searching alternative monomers with lower toxicity [100,103,104]. In particular, the toxic acrylamide inside the polyacrylamide gel dosimeter has been replaced with less harmful monomer i.e., 2-Acrylamido-2-MethylPropane Sulfonic acid (AMPS). AMPS sodium salt [100] and AMPS potassium salt [103] were used for producing these lower toxicity gels.

Various studies have analyzed the MRI response of polymer gels for possible application in radiotherapy dose mapping ([58,63] and references therein). In particular, polymer gels have been investigated for dosimetry in conformal therapy, IMRT, IMAT, IGRT, VMAT and tomotherapy [55,70,83,88,102,105,106,107,108,109,110,111,112,113,114,115,116,117,118,119,120,121,122,123,124,125,126,127,128,129,130,131,132,133,134,135], small photon fields [136,137,138], stereotactic radiosurgery [55,139,140,141,142], 60-cobalt source [143,144], brachytherapy [55,85,92,145,146,147,148,149], cyberknife [150], gamma-knife [151], low-energy X-rays [152,153], high-LET and proton therapy [55,154,155], boron capture neutron therapy [55,156,157,158,159], for interface dosimetry in the presence of tissue inhomogeneities [55,160,161], for X-ray tubes [162] and for diagnostic nuclear medicine [163]. Recently, polyacrilamide gels were proposed for dosimetry and end-to-end test for MRI-linac apparatus [164,165,166,167,168,169]. Furthermore, a polymer hydrogel foam has been proposed as a potential three-dimensional experimental dosimeter for radiation treatment verification in low-density tissue such as the lung [143,170,171,172,173,174].

**Figure 10:**
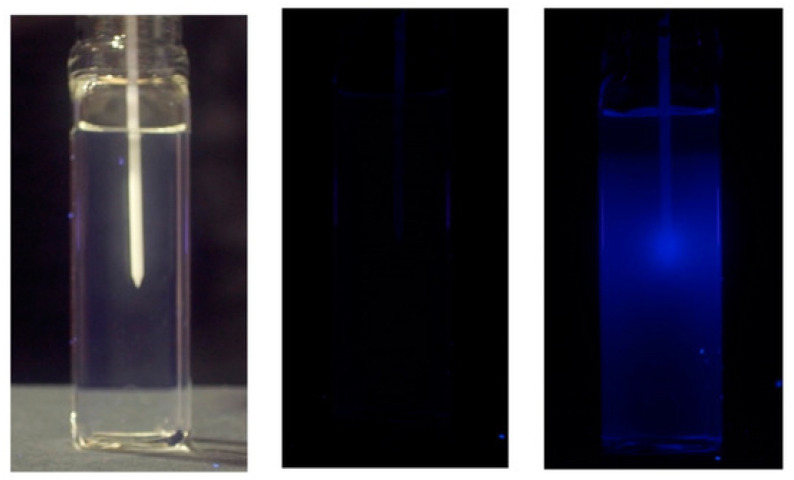
3D dosimetry of a high dose-rate seed of iridium-192 with a radio-fluorescence gel of maleimido-pyrene in tertiary-butyl acrylate. Left: Photograph under visible-light prior to seed insertion in the catheter. Middle: Photograph under UV light prior to seed insertion. Right: Photograph under UV light 3 minutes after seed insertion. Reproduced from [65].

An example of application of a polymer gel (in particular, a radio-fluorescence gel of maleimido-pyrene in tertiary-butyl acrylate) for application in high dose rate brachitherapy with a seed of iridium-192 is shown in Figure 10.

## 4. Readout Techniques

Research efforts in the field of gel dosimetry also include the development and optimization of quantitative scanning techniques. The most promising scanning techniques are magnetic resonance imaging and optical computerized tomography. For different scanning techniques, different optimal gel dosimeters can be found. Optimal gel systems for magnetic resonance imaging display a significant change in NMR relaxation time or magnetization transfer. Optical-CT techniques are an interesting alternative to the more expensive and less accessible NMR readout technique.

### 4.1. Magnetic Resonance Imaging

The first technique for dose mapping using gel dosimeters was magnetic resonance imaging (MRI) [9]. MRI is still the prevalent imaging modality since it can acquire with high spatial resolution the 3D distributions of the relaxation times of hydrogen nuclear spins. Changes in relaxation times of the nuclear spins are caused by radiation exposure and they provide a powerful tool for mapping 3D dose distributions. In the early Fricke gel systems, NMR relaxation times are affected by the change of ferrous (Fe${}^{2+}$) and ferric (Fe${}^{3+}$) ions concentrations, which are modified by the irradiation. Both these iron ions are paramagnetic species and their interactions with hydrogen nuclei significantly affect the proton relaxation times of water molecules in the vicinity of the ions. Proton relaxation times are influenced by fluctuations of the local magnetic field on the ${}^{1}$H nuclei due to the molecular motions inside the liquid [175]. These fluctuations involve spin interaction changes related to: (1) the exchange of the temporarily coordinated molecules (belonging to the so-called hydration sphere or inner coordination sphere) with the bulk water; (2) the Brownian motion of the entire ion-hydration sphere complex and (3) the relaxation of the electron spins (see [58] for more details). The influence of Fe${}^{2+}$ ions on the relaxation rates of neighboring water protons is different from that of Fe${}^{3+}$ ions because these iron ions possess different paramagnetic characteristics [58]. Irradiation induces the oxidation of Fe${}^{2+}$ ions into Fe${}^{3+}$ ions and consequently the observed NMR relaxation rates of irradiated Fricke gels is dependent on absorbed dose. Assuming a fast exchange model for describing NMR relaxation of hydrogen nuclei in which water is considered to exist in three environments (bulk, and hydrating both ferrous and ferric ions), the dependence of relaxation rates on dose can be written as follows [58]:

(16) $$R_{k}=R_{k,0}+k{\left[{\mathrm{Fe}}^{2+}\right]}_{0}R_{k,is}^{\mathrm{Fe}2+}+k\left(\frac{\partial M}{\partial D}\right)\left(R_{k,is}^{\mathrm{Fe}3+}-R_{k,is}^{\mathrm{Fe}2+}\right)D$$

where the subscript *k* is 1 for the longitudinal spin-lattice relaxation rate and 2 for the transverse spin-spin relaxation rate, respectively, $R_{k,is}^{\mathrm{Fe}2+}$ and $R_{k,is}^{\mathrm{Fe}3+}$ are the inner coordination sphere relaxation rates for ferrous and ferric ions respectively, *k* is a proportionality constant related to the molar concentration of water molecules in the solution and is the molar radiation yield (and this is equal to 1.62 µM Gy${}^{-1}$). The term $r_{k}^{D}=k\left(\frac{\partial M}{\partial D}\right)\left(R_{k,is}^{\mathrm{Fe}3+}-R_{k,is}^{\mathrm{Fe}2+}\right)$ represents the relaxation dose sensitivity (given in s${}^{-1}$ Gy${}^{-1}$) and is dependent also on the magnetic field strength.

Fricke gels show a wide dose range (up to about one hundred Gy) over which the measured radiation response of the MRI signal is linear. At higher doses, the linear response changes into a saturation trend due to the depletion of the finite amount of ferrous ions introduced in the formulation.

For magnetic fields of about 0.5 T, a good agreement is found between calculated and measured values of the longitudinal relaxation dose sensitivity (calculated value $r_{1}^{D}=0.013$ s${}^{-1}$ Gy${}^{-1}$ and measured value $r_{1}^{D}=0.0113$ s${}^{-1}$ Gy${}^{-1}$) and the transverse relaxation dose sensitivity (calculated value $r_{2}^{D}=0.0178$ s${}^{-1}$ Gy${}^{-1}$ and measured value $r_{2}^{D}=0.0121$ s${}^{-1}$ Gy${}^{-1}$). Sensitivity is dependent on the pH of the Fricke solution also because pH affects the chemical yield of the water radiolytic products and the radiochemical yield of ferrous to ferric ion conversion. The radiochemical yield can be increased by the addition of aliphatic alcohols and benzoic acid [58,176].

Fricke solutions require high doses for the radiation-induced changes to be readily observed; however, these doses can be delivered in a short time with modern LINACs. A more severe limitation is the diffusion of the radiation induced signal. In modern clinical radiations treatments, highly nonuniform, conformal dose distributions are delivered matching the often complex shape of the tumor targets. The assessment of spatial dose is of the utmost importance for the success of the therapy. However, very sharp dose gradients produce equally sharp gradients of the Fe${}^{3+}$ concentrations. These cause a significant diffusion of the ferrous and ferric ions, despite the hindrance created by the gel matrix and possibly the chelating agents added to stabilize the ion distributions. The 3D dose map is therefore blurred over time ([45,55,177,178,179,180,181] and references therein). This is why Fricke gels should be read soon after an irradiation using the fastest available imaging protocols. The ion diffusion has been very extensively studied and characterized; it was found that the diffusion coefficients of Fricke gels depend on the type of gel, the gel concentration, the temperature and other properties of the dosimeter. This is one of the main drawbacks for extensive use of Fricke gels which has pushed interest for the development and increased clinical use of polymer gels [55,182].

Polyacrylamide dosimeters may also be scanned with MRI techniques and various contrast parameters may be used to map the spatial distributions of radiation-induced polymerization [55,58,182,183]. Changes induced on the spin-lattice relaxation rates R${}_{1}$ as a consequence of irradiation are relatively small, while the variations of the spin-spin R${}_{2}$ provide a stronger dose dependent signal with a wider dynamic range. In order to describe the dependence of the relaxation rate R${}_{1}$ and R${}_{2}$ on absorbed dose, it is useful to introduce a model which considers different proton pools [58]: (1) a mobile proton pool composed of free and quasi-free protons, (2) the (poly) proton pool of a growing polymer network and (3) the (gela) proton pool of the gelatin matrix. For polyacrylamide gel dosimeters, a rapid exchange limit is assumed [175] in which the lifetimes of the various pools are short with respect to the relaxation times but still long compared to the correlation times, and it is verified. Within the rapid exchange limit, the measured relaxation curve can be considered to be monoexponential with a relaxation rate that is the weighted average of the relaxation rates of the different proton pools in the entire sample [58]:

(17) $$R_{2}=f_{mob}R_{2,mob}+f_{poly}R_{2,poly}+f_{gela}R_{2,gela}$$

with $f_{mob}$, $f_{poly}$ and $f_{gela}$ the relative fractions of protons in the mobile, polymer and gelatin pool, respectively. During irradiation, the amount of mobile hydrogen and polymer aggregates changes because of polymerization processes. In particular, as dose increases the concentration of mobile hydrogens (present in water molecules and in relatively small monomers) decreases and the polymerization yields large growing polymers in which bound hydrogens present large relaxation rates. This results in a relaxation time T${}_{2}$ reduction with increasing absorbed radiation dose. Conversely, the gelatin pool is supposed to be unaffected by the irradiation process [58]. The acquisition of R${}_{2}$ maps minimizes the effects of inhomogeneities of the radio-frequency field (B${}_{1}$ field) and of the external magnetic field (B${}_{0}$ field) more effectively than with T${}_{2}$-weighted images. The simplest way to achieve quantitative R${}_{2}$ maps is to acquire different images with spin-echo sequences changing the TE. Based on the exponential behavior of spin echo intensity with TE, the R${}_{2}$ value in each pixel can be acquired from two differently T${}_{2}$-weighted images according to the following equation,

(18) $$R_{2}\left(i,j\right)=\frac{1}{{\mathrm{TE}}_{2}-{\mathrm{TE}}_{1}}\ln\left[\frac{S_{1}\left(i,j\right)}{S_{2}\left(i,j\right)}\right]$$

where TE${}_{1}$ and TE${}_{2}$ are the two echo times and $S_{1}\left(i,j\right)$ and $S_{2}\left(i,j\right)$ are the respective signals for a given pixel. A more accurate determination may be obtained with a multiple spin echo sequence and a suitable fitting procedure [184]. Mapping of the magnetization transfer (MT) has also been investigated [185] and was recently advanced for scanning low-density gel dosimeters [58]. Furthermore, investigations on polyacrilamide gels for dosimetric purposes were performed with high field magnetic resonance microimaging [186]. Recently, a new MRI approach, based on detecting nuclear Overhauser enhancement (NOE) mediated saturation transfer effects, was adopted on MAGIC gels for 3D-dose reconstruction [187].

### 4.2. Optical Computed Tomography

Shortly after the introduction of dosimetric gels, 3D scanning with optical CT was advanced as a possible alternative to the more expensive MRI procedures [9,182]. The field is still very active, especially for application to polymer gels, and a variety of systems have been developed and described: first-generation laser beam scanners, faster fan beam laser scanners and parallel or cone beam scanners with area detectors [188]. Laser beam scanners offer good scattered-light rejection, but their operation is time consuming because of the extensive motion of pencil beam required for a complete scan. Conversely, area detector scanners are much faster as all projections are acquired simultaneously for each angle of rotation of the dosimeter sample. However, in area detection, stray light can cause artifacts, especially with polymer gels that primarily scatter light contrary to radiochromic Fricke gels that primarily absorb light. Some laser scanners incorporate stray light rejection of the scanning laser beam and replace laser translation with faster options, such as a rotating mirror that fans the beam through the sample [189,190].

A common element of various optical CT scanner designs is a tank in which the gel sample is placed and which contains a fluid whose refractive index matches that of the gels. The fluid helps keeping the desired beam paths through the sample by minimizing the effects of refraction and reflection on the sample container and gel interfaces. A variant is the design by Maryanski and Ranade [189] where a rotating mirror laser beam scanner uses the cylindrical walls of the gel container to generate parallel beam paths through a central part of the gel, without the use of a tank with matching refractive index fluid. This design has been used for the measurement of dose distributions from centrally located brachytherapy sources [191,192] and for a small central ${}^{60}$Co field [193]. However, this scanner is not ideal for external beam dosimetry as the projection data are limited to the middle third of the gel sample and this can lead to inaccurate reconstruction when significant optical attenuation occurs in the missing data regions. A more recent design utilizes a a plastic cylindrical container that provides parallel beam geometry through the gel sample for the majority of the projections and no longer requires immersion in a tank with matching refractive index fluid [194]. New algorithms were developed for 3D dose reconstruction with these scanners [195].

### 4.3. X-ray Computed Tomography

One of the factors hindering a widespread implementation and routine use of the MRI gel dosimetry technique is the limited access to MRI scanners in clinical radiation therapy settings. Even optical tomography systems are arguably specialized equipment that is not commonly available for clinical use. This motivated investigations on the use of X-ray computed tomography (CT) for performing polyacrylamide gel dosimetry. The basis for this approach is the local variation of density accompanying the radiation induced polymerization of irradiated gels. This change in density produces a low-level contrast in CT images, which can be used for practical imaging purposes. Indeed, reports of X-ray computed tomography of irradiated PAGs are present in the literature [182,196]. The dose resolution is only ∼0.5 Gy for an image thickness of 10 mm; nevertheless, CT scans were shown to provide accurate localization of high dose gradients such as those observed in stereotactic radiosurgery. A full development of this approach may lead to a relatively inexpensive and accessible way to analyze polyacrylamide dosimeters benefiting from the availability and speed of CT scanners in clinical settings [151,197,198,199,200,201].

### 4.4. Ultrasound Imaging

Ultrasonic (US) imaging was also considered for the determination of spatial dose distribution for polyacrylamide gels. This is because US imaging is of easy access, relatively rapid acquisition and low cost. The use of ultrasound imaging is possible because the polymerization induced by ionizing radiations causes variations of ultrasonic properties such as the acoustic speed of propagation, ultrasonic absorption and ultrasonic attenuation change ([202] and references therein). In particular, radiation exposure induces variations in both the elastic modulus and mass density, thus modifying ultrasound speed, as observed for PAG and MAGIC gel dosimeters [203]. Various investigations with ultrasound techniques were carried out also on PAGAT gel dosimeters [204], on a gel composed of a monomer named 2-Hydroxyl-Ethyl-Meta-Acrylate (HEMA) in the presence of gelatine as a gelling agent [205] and a MAGIC gel modified by adding formaldehyde (MAGIC-f) [206] and on a MAGIC polymer gel proprietary combination with ultrasonic soft tissue-mimicking gel [207]. Detailed studies were performed to investigate the phenomena on the basis of ultrasound features correlated with radiation-induced polymerization [202,208,209]. More recently, ultrasonic shear wave elasticity imaging (SWEI), which is a nondestructive and quantitative elasticity imaging tool, was successfully used for mapping the 3D dose distribution with a NIPAM gel [210].

Efforts have also been reported towards the development of a tomographic ultrasound system comprising a table able to translate and rotate around a suspended gel phantom, an ultrasound transducer which produces US acoustic waves and a needle hydrophone that detects transmitted or reflected US waves [202]. Images can be obtained by analyzing time difference (for time-of-flight, TOF, acquisitions) and amplitude difference (for transmission acquisitions) of the transmitted and received waves. TOF images show better image quality, whereas transmission images offer better contrast between unirradiated and irradiated regions of the gels [202].

## 5. Conclusions

Gel dosimetry has raised considerable interest for nearly two decades as it offers a promise of high resolution, tissue equivalent 3D dosimetry. Research and development are ongoing, and gel compositions are constantly improving. Extensive studies have been conducted on the chemistry of dosimetric gels and different formulations have been proposed and tried.

However, neither ferrous-sulphate-infused gels nor polyacrylamide gels have come into common clinical use. Concerns are still substantial on the spatial dose mapping accuracy of Fricke gels, mainly due to diffusion and spontaneous oxidation effects. Regarding polyacrylamide gels, these are difficult to prepare in a hospital setting as they contain toxic ingredients and often require anoxic preparation.

A deeper understanding of the physical and chemical interactions within the gels helps to optimize the compositions and formulate materials that provide improved stability, spatial integrity, temperature insensitivity, dose rate and energy independence and tissue equivalence. The recent introduction of new Fricke gel formulations based on synthetic chemicals and of less toxic ingredients for polyacrylamide gel dosimeters are motivating the continuing development of this technology.

The need for expensive MRI readout has also limited the use of gel dosimeters. The development of optical imaging techniques, such as those described in this review, have made their use in the clinical setting closer. The ultimate goal is achieving a level of spatial and dose accuracy and precision allowing the verification of clinical radiation treatment plans.

At present, no other 3D dosimeter exists that can serve as a “gold standard” and even a reference for both the spatial and dose accuracy is difficult to define. Thus, gel dosimetry investigators have defined their target as a system able to deliver the 3D analysis of a treatment plan within 1 hour, with 1 mm isotropic spatial resolution, an accuracy within 3% of the true value and 1% precision.

Overall, the challenge is formidable, but it is exactly the lack of viable solutions for the verification of the ever-more-complex radiotherapy delivery modalities that provides the strongest motivation for the ongoing research efforts in 3D gel dosimetry.

## Figures and Tables

**Table 1:** Gelation times of gels produced with different concentrations of GTA and held at different gelation temperatures. Adapted from [26].

GTA (mM)	Temperature (°C)	Gelation Time (min)
13.25	15	N/A
26.5	15	N/A
53	15	30
13.25	20	75
26.5	20	35
53	20	25
13.25	25	25
26.5	25	20
53	25	10

**Table 2:** Diffusion coefficients of the investigated PVA Fricke gels. Adapted from [26].

PVA Type	PVA Concentration	Molecular Weight	GTA Concentration	Diffusion Coefficient
	(% *w*/*v*)	(×10^3^)	(mM)	(mm^2^h^−1^)
PVA-A	10%	85–124	26.5	0.171 ± 0.012
PVA-B	10%	85–124	6.625	0.249 ± 0.029
PVA-C	10%	31–50	26.5	0.200 ± 0.018
PVA-D	10%	13–23	26.5	0.291 ± 0.033
Mw-A	10%	125	26.5	0.184 ± 0.014
Mw-B	12.50%	125	33.125	0.152 ± 0.008

**Table 3:** Comparison with other gel types and studies.

Gel Matrix	Sensitivity	Diffusion Coefficient	Spontaneous Oxidation
	(Gy^−1^)	(mm^2^ h^−1^)	(h^−1^)
PVA-GTA (Mw-B)	0.077 ± 0.001 (1s.e.)	0.152 ± 0.008 (1s.d.)	2.60 × 10^−4^
20% PVA hydrogel [46]	0.046 (uncertainty not reported)	0.140 ± 0.002	1.3 × 10^−4^
20% functionalized PVA [48]	0.014 (uncertainty not reported)	0.130 ± 0.004	–
Natural polymers (various types and concentrations)	0.065–0.075	0.3–2.2	2.3 × 10^−3^
[16,40,46,47,49]	(uncertainty reported as standard errors)		

## References

[1] UNSCEAR United Nations Scientific Committee on the Effects of Atomic Radiation Reports. https://www.unscear.org/.

[2] D’Errico F. (2006). Dosimetric issues in radiation protection of radiotherapy patients. Radiat. Prot. Dosim..

[3] Bouchard H., Seuntjens J., Carrier J.F., Kawrakow I. (2009). Ionization chamber gradient effects in nonstandard beam configurations. Med. Phys..

[4] Griessbach I., Lapp M., Bohsung J., Gademann G., Harder D. (2005). Dosimetric characteristics of a new unshielded silicon diode and its application in clinical photon and electron beams. Med. Phys..

[5] Porumb C.S., Aldosari A.H., Fuduli I., Cutajar D., Newall M., Metcalfe P., Carolan M., Lerch M.L., Perevertaylo V.L., Rosenfeld A.B. (2016). Characterisation of silicon diode arrays for dosimetry in external beam radiation therapy. IEEE Trans. Nucl. Sci..

[6] Kry S.F., Alvarez P., Cygler J.E., DeWerd L.A., Howell R.M., Meeks S., O’Daniel J., Reft C., Sawakuchi G., Yukihara E.G. (2020). AAPM TG 191: Clinical use of luminescent dosimeters: TLDs and OSLDs. Med. Phys..

[7] Devic S., Tomic N., Lewis D. (2016). Reference radiochromic film dosimetry: Review of technical aspects. Phys. Med..

[8] Oldham M. (2014). Methods and techniques for comprehensive 3D dosimetry. Adv. Med. Phys..

[9] Gore J., Kang Y. (1984). Measurement of radiation dose distributions by nuclear magnetic resonance (NMR) imaging. Phys. Med. Biol..

[10] Appleby A., Christman E., Leghrouz A. (1987). Imaging of spatial radiation dose distribution in agarose gels using magnetic resonance. Med. Phys..

[11] Gambarini G., Arrigoni S., Cantone M., Molho N., Facchielli L., Sichirollo A. (1994). Dose-response curve slope improvement and result reproducibility of ferrous-sulphate-doped gels analysed by NMR imaging. Phys. Med. Biol..

[12] Luciani A., Di Capua S., Guidoni L., Ragona R., Rosi A., Viti V. (1996). Multiexponential relaxation in Fricke agarose gels: Implications for NMR dosimetry. Phys. Med. Biol..

[13] Maryanski M.J., Gore J.C., Kennan R.P., Schulz R.J. (1993). NMR relaxation enhancement in gels polymerized and cross-linked by ionizing radiation: A new approach to 3D dosimetry by MRI. Magn. Reson. Imaging.

[14] Fong P.M., Keil D.C., Does M.D., Gore J.C. (2001). Polymer gels for magnetic resonance imaging of radiation dose distributions at normal room atmosphere. Phys. Med. Biol..

[15] Fricke H., Morse S. (1927). The chemical action of roentgen rays on dilute ferrosulphate solutions as a measure of dose. Am. J. Roentgenol. Radium Ther. Nucl. Med.

[16] Rae W.I., Willemse C.A., Lötter M.G., Engelbrecht J.S., Swarts J.C. (1996). Chelator effect on ion diffusion in ferrous-sulfate-doped gelatin gel dosimeters as analyzed by MRI. Med. Phys..

[17] Schreiner L. (2015). True 3D chemical dosimetry (gels, plastics): Development and clinical role. J. Phys. Conf. Ser..

[18] Kelly R., Jordan K., Battista J. (1998). Optical CT reconstruction of 3D dose distributions using the ferrous–benzoic–xylenol (FBX) gel dosimeter. Med. Phys..

[19] Oldham M., Siewerdsen J.H., Shetty A., Jaffray D.A. (2001). High resolution gel-dosimetry by optical-CT and MR scanning. Med. Phys..

[20] Olsson L.E., Petersson S., Ahlgren L., Mattsson S. (1989). Ferrous sulphate gels for determination of absorbed dose distributions using MRI technique: Basic studies. Phys. Med. Biol..

[21] Bero M., Gilboy W., Glover P., El-Masri H. (2000). Tissue-equivalent gel for non-invasive spatial radiation dose measurements. Nucl. Instrum. Methods Phys. Res. Sect. B Beam Interact. Mater. Atoms.

[22] Hazle J.D., Hefner L., Nyerick C., Wilson L., Boyer A. (1991). Dose-response characteristics of a ferrous-sulphate-doped gelatin system for determining radiation absorbed dose distributions by magnetic resonance imaging (Fe MRI). Phys. Med. Biol..

[23] Mansur H.S., Sadahira C.M., Souza A.N., Mansur A.A. (2008). FTIR spectroscopy characterization of poly (vinyl alcohol) hydrogel with different hydrolysis degree and chemically crosslinked with glutaraldehyde. Mater. Sci. Eng. C.

[24] D’Errico F., Lazzeri L., Dondi D., Mariani M., Marrale M., Souza S.O., Gambarini G. (2017). Novel GTA-PVA Fricke gels for three-dimensional dose mapping in radiotherapy. Radiat. Meas..

[25] Marini A., Lazzeri L., Cascone M.G., Ciolini R., Tana L., d’Errico F. (2017). Fricke gel dosimeters with low-diffusion and high-sensitivity based on a chemically cross-linked PVA matrix. Radiat. Meas..

[26] Lazzeri L., Marini A., Cascone M.G., d’Errico F. (2019). Dosimetric and chemical characteristics of Fricke gels based on PVA matrices cross-linked with glutaraldehyde. Phys. Med. Biol..

[27] Prentø P. (1995). Glutaraldehyde for electron microscopy: A practical investigation of commercial glutaraldehydes and glutaraldehyde-storage conditions. Histochem. J..

[28] Polysciences Inc. (2012). Glutaraldehyde Fixative Technical Data Sheet 883.

[29] Rasmussen K.E., Albrechtsen J. (1974). Glutaraldehyde. The influence of pH, temperature, and buffering on the polymerization rate. Histochemistry.

[30] GmbH C. (1999). ® Mowiol Polyvinyl Alcohol Technical Data Sheet.

[31] Shin J., Kim Y., Lee K., Lim Y.M., Nho Y.C. (2008). Significant effects of sodium acetate, an impurity present in poly (vinyl alcohol) solution on the radiolytic formation of silver nanoparticle. Radiat. Phys. Chem..

[32] Shin J., Kim Y., Lim Y.M., Nho Y.C. (2008). Removal of sodium acetate in poly (vinyl alcohol) and its quantification by 1H NMR spectroscopy. J. Appl. Polym. Sci..

[33] Del Rocio Bernal Zamorano M. (2018). Development and Characterization of Novel Radiochromic Dosimeters for X-Rays and UV Radiations. Ph.D. Thesis.

[34] Liosi G., Dondi D., Vander Griend D., Lazzaroni S., D’Agostino G., Mariani M. (2017). Fricke-gel dosimeter: Overview of Xylenol Orange chemical behavior. Radiat. Phys. Chem..

[35] Kron T., Metcalfe P., Pope J. (1993). Investigation of the tissue equivalence of gels used for NMR dosimetry. Phys. Med. Biol..

[36] Gallo S., Artuso E., Brambilla M.G., Gambarini G., Lenardi C., Monti A.F., Torresin A., Pignoli E., Veronese I. (2019). Characterization of radiochromic poly (vinyl-alcohol)–glutaraldehyde Fricke gels for dosimetry in external x-ray radiation therapy. J. Phys. D. Appl. Phys..

[37] Schulz R., DeGuzman A., Nguyen D., Gore J. (1990). Dose-response curves for Fricke-infused agarose gels as obtained by nuclear magnetic resonance. Phys. Med. Biol..

[38] Olsson L.E., Westrin B.A., Fransson A., Nordell B. (1992). Diffusion of ferric ions in agarose dosimeter gels. Phys. Med. Biol..

[39] Harris P., Piercy A., Baldock C. (1996). A method for determining the diffusion coefficient in Fe (II/III) radiation dosimetry gels using finite elements. Phys. Med. Biol..

[40] Kron T., Jonas D., Pope J.M. (1997). Fast T1 imaging of dual gel samples for diffusion measurements in NMR dosimetry gels. Magn. Reson. Imaging.

[41] Pedersen T.V., Olsen D.R., Skretting A. (1997). Measurement of the ferric diffusion coefficient in agarose and gelatine gels by utilization of the evolution of a radiation induced edge as reflected in relaxation rate images. Phys. Med. Biol..

[42] De Pasquale F., Sebastiani G., Egger E., Guidoni L., Luciani A.M., Marzola P., Manfredi R., Pacilio M., Piermattei A., Viti V. (2000). Bayesian estimation of relaxation times T1 in MR images of irradiated Fricke-agarose gels. Magn. Reson. Imaging.

[43] Baldock C., Harris P., Piercy A., Healy B. (2001). Experimental determination of the diffusion coefficient in two-dimensions in ferrous sulphate gels using the finite element method. Australas. Phys. Eng. Sci. Med..

[44] Tseng Y., Chu W., Chung W., Guo W., Kao Y.H., Wang J., Huang S.C. (2002). The role of dose distribution gradient in the observed ferric ion diffusion time scale in MRI-Fricke-infused gel dosimetry. Magn. Reson. Imaging.

[45] Marrale M., Collura G., Gallo S., Nici S., Tranchina L., Abbate B.F., Marineo S., Caracappa S., d’Errico F. (2017). Analysis of spatial diffusion of ferric ions in PVA-GTA gel dosimeters through magnetic resonance imaging. Nucl. Instrum. Methods Phys. Res. Sect. B Beam Interact. Mater. Atoms.

[46] Chu K., Jordan K., Battista J., Van Dyk J., Rutt B. (2000). Polyvinyl alcohol-Fricke hydrogel and cryogel: Two new gel dosimetry systems with low Fe3+ diffusion. Phys. Med. Biol..

[47] Healy B., Brindha S., Zahmatkesh M., Baldock C. (2004). Characterisation of the ferrous-xylenol orange-gelatin(FXG) gel dosimeter. J. Phys. Conf. Ser..

[48] Smith S., Masters K.S., Hosokawa K., Blinco J., Crowe S., Kairn T., Trapp J. (2015). A reduction of diffusion in PVA Fricke hydrogels. J. Phys. Conf. Ser..

[49] Davies J., Baldock C. (2008). Sensitivity and stability of the Fricke–gelatin–xylenol orange gel dosimeter. Radiat. Phys. Chem..

[50] Lazzaroni S., Liosi G., Mariani M., Dondi D. (2020). An innovative Fe3+ selective ligand for Fricke-gel dosimeter. Radiat. Phys. Chem..

[51] Alves A.V.S., de Almeida W.S., Sussuchi E.M., Lazzeri L., d’Errico F., de Souza S.O. (2018). Investigation of chelating agents/ligands for Fricke gel dosimeters. Radiat. Phys. Chem..

[52] Eyadeh M.M., Rabaeh K.A., Hailat T.F., Al-Shorman M.Y., Aldweri F.M., Kanan H.M., Awad S.I. (2018). Investigation of a novel chemically cross-linked fricke-Methylthymol blue-synthetic polymer gel dosimeter with glutaraldehyde cross-linker. Radiat. Meas..

[53] Maeyama T., Fukunishi N., Ishikawa K.L., Fukasaku K., Fukuda S. (2016). Radiological properties of nanocomposite Fricke gel dosimeters for heavy ion beams. J. Radiat. Res..

[54] Taño J., Hayashi S., Hirota S., Gonzales C., Yasuda H. (2019). Development of a reusable PVA-GTA-I gel dosimeter for 3D radiation dose assessments. J. Phys. Conf. Ser..

[55] Baldock C., De Deene Y., Doran S., Ibbott G., Jirasek A., Lepage M., McAuley K., Oldham M., Schreiner L. (2010). Polymer gel dosimetry. Phys. Med. Biol..

[56] Baldock C., Burford R., Billingham N., Wagner G., Patval S., Badawi R.D., Keevil S. (1998). Experimental procedure for the manufacture and calibration of polyacrylamide gel (PAG) for magnetic resonance imaging (MRI) radiation dosimetry. Phys. Med. Biol..

[57] Magee J.L., Chatterjee A., Freeman G.R. (1987). Chapter 4: Track reactions of radiation chemistry. Kinetics of Nonhomogeneous Processes.

[58] De Deene Y. (2020). Chapter 9: Gel-based Radiation Dosimetry Using Quantitative MRI. New Developments in NMR.

[59] BrahimiMoussa S., Benamar M.E.A., LounisMokrani Z. (2020). Characterization of the chemical and structural modifications induced by gamma rays on the MAGIC polymer gel. Radiat. Phys. Chem..

[60] Chacón D., Vedelago J., Strumia M., Valente M., Mattea F. (2019). Raman spectroscopy as a tool to evaluate oxygen effects on the response of polymer gel dosimetry. Appl. Radiat. Isot..

[61] Magugliani G., Liosi G., Tagliabue D., Mossini E., Negrin M., Mariani M. (2018). Characterization of PAGAT dose response upon different irradiation conditions. Radiat. Eff. Defects Solids.

[62] Kozicki M., Jaszczak M., Maras P., Dudek M., Cłapa M. (2017). On the development of a VIPARnd radiotherapy 3D polymer gel dosimeter. Phys. Med. Biol..

[63] Farhood B., Geraily G., Abtahi S.M.M. (2019). A systematic review of clinical applications of polymer gel dosimeters in radiotherapy. Appl. Radiat. Isot..

[64] Cheng K.Y., Hsieh L.L., Shih C.T. (2016). A comprehensive evaluation of NIPAM polymer gel dosimeters on three orthogonal planes and temporal stability analysis. PLoS ONE.

[65] Warman J.M., De Haas M.P., Luthjens L.H., Denkova A.G., Yao T. (2018). A radio-fluorogenic polymer-gel makes fixed fluorescent images of complex radiation fields. Polymers.

[66] Low D.A., Mutic S., Dempsey J.F., Gerber R.L., Bosch W.R., Perez C.A., Purdy J.A. (1998). Quantitative dosimetric verification of an IMRT planning and delivery system. Radiother. Oncol..

[67] Cho K., Cho S., Lee S., Lee S., Min C., Kim Y., Moon S., Kim E., Chang A., Kwon S. (2012). Dose responses in a normoxic polymethacrylic acid gel dosimeter using optimal CT scanning parameters. Nucl. Instrum. Methods Phys. Res. Sect. A Accel. Spectrometers, Detect. Assoc. Equip..

[68] Jirasek A., Hilts M., McAuley K. (2010). Polymer gel dosimeters with enhanced sensitivity for use in x-ray CT polymer gel dosimetry. Phys. Med. Biol..

[69] Khadem-Abolfazli M., Mahdavi M., Mahdavi S., Ataei G. (2013). Dose enhancement effect of gold nanoparticles on MAGICA polymer gel in mega voltage radiation therapy. Int. J. Radiat. Res..

[70] Silveira M., Pavoni J.F., Baffa O. (2017). Three-dimensional quality assurance of IMRT prostate plans using gel dosimetry. Phys. Med..

[71] Abtahi S.M.M., Abandansari H.S. (2017). Polymer gel dosimeters with PVA–GA matrix. Australas. Phys. Eng. Sci. Med..

[72] Abtahi S., Aghamiri S., Khalafi H. (2014). Optical and MRI investigations of an optimized acrylamide-based polymer gel dosimeter. J. Radioanal. Nucl. Chem..

[73] Pavoni J., Baffa O. (2012). An evaluation of dosimetric characteristics of MAGIC gel modified by adding formaldehyde (MAGIC-f). Radiat. Meas..

[74] Abtahi S. (2016). Characteristics of a novel polymer gel dosimeter formula for MRI scanning: Dosimetry, toxicity and temporal stability of response. Phys. Med..

[75] Hayashi S.i., Yoshioka M., Usui S., Haneda K., Kondo T., McAuley K.B., Tominaga T. (2010). A study on the role of gelatin in methacrylic-acid-based gel dosimeters. Radiat. Phys. Chem..

[76] Farhood B., Geraily G., Abtahi S.M.M., Ghorbani M., Mehdikhani M. (2018). Evaluation of dose rate and photon energy dependence of PASSAG polymer gel dosimeter. J. Radioanal. Nucl. Chem..

[77] Shih T.Y., Yen T.H., Liu Y.L., Luzhbin D., Wu J. (2017). Evaluation of characteristics of high-energy electron beams using N-isopropyl-acrylamide gel dosimeter. Radiat. Phys. Chem..

[78] Anaraki V., Abtahi S.M.M., Farhood B., Ejtemai-fard M. (2018). A novel method for increasing the sensitivity of NIPAM polymer gel dosimeter. Radiat. Phys. Chem..

[79] Hayashi S.i., Kawamura H., Usui S., Tominaga T. (2018). Influence of magnesium chloride on the dose–response of polyacrylamide-type gel dosimeters. Radiol. Phys. Technol..

[80] Khosravi H., Rahmani F., Hashemi B. (2016). Gel dosimetry: The effect of gold nanoparticles on the dose enhancement in the external radiation therapy. Nanomed. Res. J..

[81] Santibáñez M., Guillen Y., Chacón D., Figueroa R., Valente M. (2018). Feasibility of dose enhancement assessment: Preliminary results by means of Gd-infused polymer gel dosimeter and Monte Carlo study. Appl. Radiat. Isot..

[82] Santibáñez M., Fuentealba M., Vedelago J., Chacón D., Mattea F., Valente M. (2021). Experimental characterization and Monte Carlo simulations of the dose enhancement on the millimeter scale of PAGAT infused with Gadolinium. Radiat. Phys. Chem..

[83] Mustaqim A., Yahaya N., Razak N., Zin H. (2020). The dose enhancement of MAGAT gel dosimeter doped with zinc oxide at 6 MV photon beam. Radiat. Phys. Chem..

[84] Farahani S., Alam N.R., Haghgoo S., Shirazi A., Geraily G., Gorji E., Kavousi N. (2020). The effect of bismuth nanoparticles in kilovoltage and megavoltage radiation therapy using magnetic resonance imaging polymer gel dosimetry. Radiat. Phys. Chem..

[85] Ko S.Y., Kwon S.I., Chun J., Shin H.S., Chang S.K., Im J.H., Lee J.I. (2019). Effect of Gold Nanoparticles on Dose Enhancement in Brachytherapy Using a Polymer Gel Dosimeter. J. Korean Phys. Soc..

[86] Rajaee A., Wang S., Zhao L., Liu Y. (2019). Gel dosimetry measurement of dose enhancement bismuth-based nanoparticles in radiation therapy. J. Phys. Conf. Ser..

[87] Shih T.Y., Hsieh B.T., Yen T.H., Lin F.Y., Wu J. (2019). Sensitivity enhancement of methacrylic acid gel dosimeters by incorporating iodine for computed tomography scans. Phys. Med..

[88] Farahani S., Alam N.R., Haghgoo S., Khoobi M., Geraily G., Gorji E. (2019). Dosimetry and Radioenhancement Comparison of Gold Nanoparticles in Kilovoltage and Megavoltage Radiotherapy using MAGAT Polymer Gel Dosimeter. J. Biomed. Phys. Eng..

[89] Behrouzkia Z., Zohdiaghdam R., Khalkhali H., Mousavi F. (2019). Evaluation of Gold Nanoparticle Size Effect on Dose Enhancement Factor in Megavoltage Beam Radiotherapy Using MAGICA Polymer Gel Dosimeter. J. Biomed. Phys. Eng..

[90] Samuel E.J.J., Srinivasan K., Poopathi V. (2017). Radiation dose enhancement of gold nanoparticle on different polymer gel dosimeters. J. Phys. Conf. Ser..

[91] Sabbaghizadeh R., Shamsudin R., Deyhimihaghighi N., Sedghi A. (2017). Enhancement of dose response and nuclear magnetic resonance image of PAGAT polymer gel dosimeter by adding silver nanoparticles. PLoS ONE.

[92] Khosravi H., Hashemi B., Rahmani F., Ebadi A. (2016). Investigation of the gold nanoparticles effects on the prostate dose distribution in brachytherapy: Gel dosimetry and Monte Carlo method. J. Contemp. Brachytherapy.

[93] Khan M., Heilemann G., Lechner W., Georg D., Berg A.G. (2019). Basic Properties of a New Polymer Gel for 3D-Dosimetry at High Dose-Rates Typical for FFF Irradiation Based on Dithiothreitol and Methacrylic Acid (MAGADIT): Sensitivity, Range, Reproducibility, Accuracy, Dose Rate Effect and Impact of Oxygen Scavenger. Polymers.

[94] Waldenberg C., Hauer A.K., Gustafsson C., Ceberg S. (2017). Dose integration and dose rate characteristics of a NiPAM polymer gel MRI dosimeter system. J. Phys. Conf. Ser..

[95] Aliasgharzadeh A., Mohammadi A., Farhood B., Anaraki V., Mohseni M., Moradi H. (2020). Improvement of the sensitivity of PASSAG polymer gel dosimeter by urea. Radiat. Phys. Chem..

[96] Abtahi S.M.M., Anaraki V., Farhood B., Mahdavi S.R. (2020). Assessment of photon energy and dose rate dependence of U-NIPAM polymer gel dosimeter. Radiat. Phys. Chem..

[97] Farhood B., Mohammadi ASL K., Sarvizadeh M., Aliasgharzadeh A. (2020). Dosimetric evaluation of PASSAG-U polymer gel dosimeter: Dependence of dose rate and photon energy. J. X-ray Sci. Technol..

[98] Sathiyaraj P., Samuel J.J. (2018). Dose rate and energy dependence study of methacrylic acid gelatin tetrakis (hydroxymethyl) phosphonium chloride gel with flattened and unflattened photon beams. J. Cancer Res. Ther..

[99] Sellakumar P., Samuel E.J.J. (2010). Study on energy dependence of PAGAT polymer gel dosimeter evaluated using X-ray CT. Radiat. Meas..

[100] Farhood B., Abtahi S.M.M., Geraily G., Ghorbani M., Mahdavi S.R., Zahmatkesh M.H. (2018). Dosimetric characteristics of PASSAG as a new polymer gel dosimeter with negligible toxicity. Radiat. Phys. Chem..

[101] Farajollahi A., PAK F., Horsfield M., Myabi Z. (2014). The basic radiation properties of the N-isopropylacrylamide based polymer gel dosimeter. Int. J. Radiat. Res..

[102] Ghaseminejad S., Mesbahi A., Khajeali A., Farajollahi A.R. (2017). Dosimetric evaluation of small IMRT beamlets in the presence of bone inhomogeneity using NIPAM polymer gel and Monte Carlo simulation. Radiat. Meas..

[103] Rashidi A., Abtahi S.M.M., Saeedzadeh E., Akbari M.E. (2020). A new formulation of polymer gel dosimeter with reduced toxicity: Dosimetric characteristics and radiological properties. Z. Fur Med. Phys..

[104] Abtahi S.M.M., Pourghanbari M. (2018). A new less toxic polymer gel dosimeter: Radiological characteristics and dosimetry properties. Phys. Med..

[105] Yao C.H., Chang T.H., Tsai M.J., Lai Y.C., Chen Y.A., Chang Y.J., Chen C.H. (2017). Dose verification of volumetric modulation arc therapy by using a NIPAM gel dosimeter combined with a parallel-beam optical computed tomography scanner. J. Radioanal. Nucl. Chem..

[106] Kozicki M., Berg A., Maras P., Jaszczak M., Dudek M. (2020). Clinical radiotherapy application of N-vinylpyrrolidone-containing 3D polymer gel dosimeters with remote external MR-reading. Phys. Med..

[107] Chou Y.H., Lu Y.C., Peng S.L., Lee S.C., Hsieh L.L., Shih C.T. (2020). Evaluation of the dose distribution of tomotherapy using polymer gel dosimeters and optical computed tomography with ring artifact correction. Radiat. Phys. Chem..

[108] Abtahi S.M.M., Langaroodi R.K.S., Akbari M.E. (2020). Dose distribution verification in intraoperative radiation therapy using an N-isopropyl acrylamide-based polymer gel dosimeter. J. Radioanal. Nucl. Chem..

[109] Venning A., Chandroth M.M., Chick B., Waller B., Morgan C. (2019). Investigation of lung tumour peripheral doses using normoxic polymer gel and film dosimetry techniques. J. Phys. Conf. Ser..

[110] Jaszczak M., Maras P., Kozicki M. (2020). Characterization of a new N-vinylpyrrolidone-containing polymer gel dosimeter with Pluronic F-127 gel matrix. Radiat. Phys. Chem..

[111] Walg Y.P., Silveira M., Eafergan N., Krutman Y., Baffa O., Berman A., Orion I. (2020). Characterization of novel polydiacetylene gel dosimeter for radiotherapy. Biomed. Phys. Eng. Express.

[112] Pappas E., Maris T. (2020). Polymer gel 3D dosimetry in radiotherapy. Z. Fur Med. Phys..

[113] Moftah B., Basfar A.A., Almousa A.A., Al Kafi A.M., Rabaeh K.A. (2020). Novel 3D polymer gel dosimeters based on N-(3-Methoxypropyl) acrylamide (NMPAGAT) for quality assurance in radiation oncology. Radiat. Meas..

[114] Adliene D., Urbonavicius B.G., Laurikaitiene J., Puiso J. (2020). New application of polymer gels in medical radiation dosimetry: Plasmonic sensors. Radiat. Phys. Chem..

[115] Lee M., Noh S., Yoon K., Lee S.W., Yoon S.M., Jung J., Jeong C., Kwak J. (2020). Feasibility Study of Polymer Gel Dosimetry Using a 3D Printed Phantom for Liver Cancer Radiotherapy. J. Korean Phys. Soc..

[116] Santos C.J., Santos W.S., Perini A.P., Valeriano C.C., Belinato W., Caldas L.V., Neves L.P. (2020). Evaluation of polymer gels using Monte Carlo simulations. Radiat. Phys. Chem..

[117] Yao C.H., Chang T.H., Su C.T., Lai Y.C., Hsu S.M., Chen C.H., Chang Y.J. (2019). A study of dose verification and comparison for complex irradiation field with high dose rate radiation by using a 3D N-isopropylacrylamide gel dosimeter. J. Radioanal. Nucl. Chem..

[118] Abtahi S.M.M. (2019). Response overshoot: A challenge for the application of polymer gel dosimeters. J. Radioanal. Nucl. Chem..

[119] Kozicki M., Jaszczak M., Kwiatos K., Maras P., Kadlubowski S., Wach R., Dudek M. (2019). Three-dimensional radiochromic and polymer gel dosimeters with Pluronic F-127 matrix–a review of current research. J. Phys. Conf. Ser..

[120] Vahabi S., Zafarghandi M.S., Bahreinipour M. (2019). Monte Carlo simulation of some gel dosimeters’ behaviour in photonic radiation field: Research on attenuation coefficient. J. Instrum..

[121] Yao C.H., Chang T.H., Lin C.C., Lai Y.C., Chen C.H., Chang Y.J. (2019). Three-dimensional dose comparison of flattening filter (FF) and flattening filter-free (FFF) radiation therapy by using NIPAM gel dosimetry. PLoS ONE.

[122] Rabaeh K.A., Al-Ajaleen M.S., Abuzayed M.H., Aldweri F.M., Eyadeh M.M. (2019). High dose sensitivity of N-(isobutoxymethyl) acrylamide polymer gel dosimeters with improved monomer solubility using acetone co-solvent. Nucl. Instrum. Methods Phys. Res. Sect. B Beam Interact. Mater. Atoms.

[123] Mann P., Schwahofer A., Karger C. (2019). Absolute dosimetry with polymer gels—A TLD reference system. Phys. Med. Biol..

[124] Jaszczak M., Wach R., Maras P., Dudek M., Kozicki M. (2018). Substituting gelatine with Pluronic F-127 matrix in 3D polymer gel dosimeters can improve nuclear magnetic resonance, thermal and optical properties. Phys. Med. Biol..

[125] Khezerloo D., Nedaie H.A., Takavar A., Zirak A., Farhood B., Banaee N., Alidokht E. (2018). Dosimetric properties of new formulation of PRESAGE^®^ with tin organometal catalyst: Development of sensitivity and stability to megavoltage energy. J. Cancer Res. Ther..

[126] Lee H.J., Roed Y., Venkataraman S., Carroll M., Ibbott G.S. (2017). Investigation of magnetic field effects on the dose–response of 3D dosimeters for magnetic resonance–image guided radiation therapy applications. Radiother. Oncol..

[127] Khezerloo D., Nedaie H.A., Takavar A., Zirak A., Farhood B., Movahedinejhad H., Banaee N., Ahmadalidokht I., Knuap C. (2017). PRESAGE^®^ as a solid 3-D radiation dosimeter: A review article. Radiat. Phys. Chem..

[128] Takanashi T., Kawamura H., Fukasaku K., Sahade D.A., Hamada T. (2017). A comparison of the dose–response behavior of AQUAJOINT^®^-based polymer gel and PAGAT gel dosimeters measured using Optical CT and MRI. J. Phys. Conf. Ser..

[129] Roed Y., Ding Y., Wen Z., Wang J., Pinsky L., Ibbott G. (2017). The potential of polymer gel dosimeters for 3D MR-IGRT quality assurance. J. Phys. Conf. Ser..

[130] Hsieh L.L., Shieh J.I., Wei L.J., Wang Y.C., Cheng K.Y., Shih C.T. (2017). Polymer gel dosimeters for pretreatment radiotherapy verification using the three-dimensional gamma evaluation and pass rate maps. Phys. Med..

[131] Maynard E., Hilts M., Heath E., Jirasek A. (2017). Evaluation of accuracy and precision in polymer gel dosimetry. Med. Phys..

[132] Mann P., Witte M., Moser T., Lang C., Runz A., Johnen W., Berger M., Biederer J., Karger C. (2016). 3D dosimetric validation of motion compensation concepts in radiotherapy using an anthropomorphic dynamic lung phantom. Phys. Med. Biol..

[133] Rabaeh K.A., Basfar A.A., Almousa A.A., Devic S., Moftah B. (2017). New normoxic N-(Hydroxymethyl) acrylamide based polymer gel for 3D dosimetry in radiation therapy. Phys. Med..

[134] Warmington L.L., Gopishankar N., Broadhurst J.H., Watanabe Y. (2016). Polymer gel dosimetry for measuring the dose near thin high-Z materials irradiated with high energy photon beams. Med. Phys..

[135] Gopishankar N., Warmington L., Watanabe Y. (2021). Evaluation of two calibration methods for MRI-based polymer gel dosimetry. Appl. Radiat. Isot..

[136] Parwaie W., Geraily G., Shirazi A., Yarahmadi M., Shakeri A., Ardekani M.A. (2020). Evaluation of lung heterogeneity effects on dosimetric parameters in small photon fields using MAGIC polymer gel, Gafchromic film, and Monte Carlo simulation. Appl. Radiat. Isot..

[137] Parwaie W., Yarahmadi M., Nedaie H., Zahmatkesh M., Barati A., Afkhami M. (2021). Evaluation of MRI-based MAGIC polymer gel dosimeter in small photon fields. Int. J. Radiat. Res..

[138] Luthjens L.H., Yao T., Warman J.M. (2018). A polymer-gel eye-phantom for 3D fluorescent imaging of millimetre radiation beams. Polymers.

[139] Watanabe Y., Perera G.M., Mooij R.B. (2002). Image distortion in MRI-based polymer gel dosimetry of gamma knife stereotactic radiosurgery systems. Med Phys..

[140] Papagiannis P., Karaiskos P., Kozicki M., Rosiak J., Sakelliou L., Sandilos P., Seimenis I., Torrens M. (2005). Three-dimensional dose verification of the clinical application of gamma knife stereotactic radiosurgery using polymer gel and MRI. Phys. Med. Biol..

[141] Karaiskos P., Petrokokkinos L., Tatsis E., Angelopoulos A., Baras P., Kozicki M., Papagiannis P., Rosiak J., Sakelliou L., Sandilos P. (2005). Dose verification of single shot gamma knife applications using VIPAR polymer gel and MRI. Phys. Med. Biol..

[142] Han Z., Hacker F., Killoran J., Kukluk J., Aizer A., Zygmanski P. (2019). Optimization of MLC parameters for TPS calculation and dosimetric verification: Application to single isocenter radiosurgery of multiple brain lesions using VMAT. Biomed. Phys. Eng. Express.

[143] Gholami M., Shahbazi-Gahrouei D. (2018). The effect of gel homogeneity on dose response in a low-density polymer gel dosimeter for radiation therapy. J. Cancer Res. Ther..

[144] Sathiyaraj P., Samuel E.J.J. (2018). Application of bi-nanoparticle on dose enhancement effect in two different polymer gel dosimeter using spectrophotometer. J. Cancer Res. Ther..

[145] Gifford K.A., Horton Jr J.L., Jackson E.F., Steger III T.R., Heard M.P., Mourtada F., Lawyer A.A., Ibbott G.S. (2005). Comparison of Monte Carlo calculations around a Fletcher Suit Delclos ovoid with radiochromic film and normoxic polymer gel dosimetry. Med. Phys..

[146] Marques T., Fernandes J., Barbi G., Nicolucci P., Baffa O. (2009). MAGIC with formaldehyde applied to dosimetry of HDR brachytherapy source. J. Phys. Conf. Ser..

[147] Watanabe Y., Mizukami S., Eguchi K., Maeyama T., Hayashi S.i., Muraishi H., Terazaki T., Gomi T. (2019). Dose distribution verification in high-dose-rate brachytherapy using a highly sensitive normoxic N-vinylpyrrolidone polymer gel dosimeter. Phys. Med..

[148] Tachibana H., Watanabe Y., Mizukami S., Maeyama T., Terazaki T., Uehara R., Akimoto T. (2020). End-to-end delivery quality assurance of computed tomography–based high-dose-rate brachytherapy using a gel dosimeter. Brachytherapy.

[149] Adinehvand K., Rahatabad F.N. (2018). Monte-Carlo based assessment of MAGIC, MAGICAUG, PAGATUG and PAGATAUG polymer gel dosimeters for ovaries and uterus organ dosimetry in brachytherapy, nuclear medicine and Tele-therapy. Comput. Methods Programs Biomed..

[150] Al Kafi M.A., Al Moussa A., Yousof M.F.M., Maryański M.J., Moftah B. (2021). Performance of a new commercial high-definition 3D patient specific quality assurance system for CyberKnife robotic radiotherapy and radiosurgery. Radiat. Meas..

[151] Jaselskė E., Adlienė D., Rudžianskas V., Korobeinikova E., Radžiūnas A. (2020). Application of polymer dose gels for millimeter scale target/tumor pre-treatment immitation using gamma knife facility. Nucl. Instrum. Methods Phys. Res. Sect. B Beam Interact. Mater. Atoms.

[152] Boudou C., Biston M.C., Corde S., Adam J.F., Ferrero C., Estève F., Elleaume H. (2004). Synchrotron stereotactic radiotherapy: Dosimetry by Fricke gel and Monte Carlo simulations. Phys. Med. Biol..

[153] Maeyama T., Ishida Y., Kudo Y., Fukasaku K., Ishikawa K.L., Fukunishi N. (2018). Polymer gel dosimeter with AQUAJOINT^®^ as hydrogel matrix. Radiat. Phys. Chem..

[154] Gustavsson H., Bäck S.Å.J., Medin J., Grusell E., Olsson L.E. (2004). Linear energy transfer dependence of a normoxic polymer gel dosimeter investigated using proton beam absorbed dose measurements. Phys. Med. Biol..

[155] Hillbrand M., Landry G., Ebert S., Dedes G., Pappas E., Kalaitzakis G., Kurz C., Würl M., Englbrecht F., Dietrich O. (2019). Gel dosimetry for three dimensional proton range measurements in anthropomorphic geometries. Z. Fur Med. Phys..

[156] Bavarnegin E., Khalafi H., Sadremomtaz A., Kasesaz Y., Khajeali A. (2017). Investigation of dose distribution in mixed neutron-gamma field of boron neutron capture therapy using N-isopropylacrylamide gel. Nucl. Eng. Technol..

[157] Khajeali A., Khodadadi R., Kasesaz Y., Horsfield M., Farajollahi A.R. (2017). Measurement of dose distribution from treatment of shallow brain tumors in BNCT by NIPAM polymer gel. Prog. Nucl. Energy.

[158] Khajeali A., Farajollahi A.R., Kasesaz Y., Khodadadi R., Khalili A., Naseri A. (2015). Capability of NIPAM polymer gel in recording dose from the interaction of 10B and thermal neutron in BNCT. Appl. Radiat. Isot..

[159] Khajeali A., Farajollahi A.R., Khodadadi R., Kasesaz Y., Khalili A. (2015). Role of gel dosimeters in boron neutron capture therapy. Appl. Radiat. Isot..

[160] Love P., Evans P., Leach M., Webb S. (2003). Polymer gel measurement of dose homogeneity in the breast: Comparing MLC intensity modulation with standard wedged delivery. Phys. Med. Biol..

[161] Jirasek A., Marshall J., Mantella N., Diaco N., Maynard E., Teke T., Hilts M. (2020). Linac-integrated kV-cone beam CT polymer gel dosimetry. Phys. Med. Biol..

[162] Mattea F., Chacón D., Vedelago J., Valente M., Strumia M.C. (2015). Polymer gel dosimeter based on itaconic acid. Appl. Radiat. Isot..

[163] Bourbia N., Mansour K. (2016). Use of magic gel for diagnostic nuclear medicine dosimetry. Acta Phys. Pol. A.

[164] Ibbott G.S., Roed Y., Lee H., Alqathami M., Wang J., Pinsky L., Blencowe A. (2016). Gel dosimetry enables volumetric evaluation of dose distributions from an MR-guided linac. AIP Conf. Proc..

[165] De Deene Y., Wheatley M., Dong B., Roberts N., Jelen U., Waddington D., Liney G. (2020). Towards real-time 4D radiation dosimetry on an MRI-Linac. Phys. Med. Biol..

[166] Baldock C., Karger C.P., Zaidi H. (2020). Gel dosimetry provides the optimal end-to-end quality assurance dosimetry for MR-linacs. Med. Phys..

[167] Maraghechi B., Gach H.M., Setianegara J., Yang D., Li H.H. (2020). Dose uncertainty and resolution of polymer gel dosimetry using an MRI guided radiation therapy system’s onboard 0.35 T scanner. Phys. Med..

[168] Dorsch S., Mann P., Elter A., Runz A., Spindeldreier C.K., Klüter S., Karger C.P. (2019). Measurement of isocenter alignment accuracy and image distortion of an 0.35 T MR-Linac system. Phys. Med. Biol..

[169] Dorsch S., Mann P., Lang C., Haering P., Runz A., Karger C. (2018). Feasibility of polymer gel-based measurements of radiation isocenter accuracy in magnetic fields. Phys. Med. Biol..

[170] De Deene Y., Vergote K., Claeys C., De Wagter C. (2006). Three dimensional radiation dosimetry in lung-equivalent regions by use of a radiation sensitive gel foam: Proof of principle. Med. Phys..

[171] De Deene Y., Vandecasteele J., Vercauteren T. (2013). Low-density polymer gel dosimeters for 3D radiation dosimetry in the thoracic region: A preliminary study. J. Phys. Conf. Ser..

[172] Shahbazi-Gahrouei D., Gholami M., Pourfallah T.A., Keshtkar M. (2015). Does nitrogen gas bubbled through a low density polymer gel dosimeter solution affect the polymerization process?. Adv. Biomed. Res..

[173] Gharehaghaji N., Dadgar H.A. (2018). Dosimetric verification of small fields in the lung using lung-equivalent polymer gel and Monte Carlo simulation. J. Cancer Res. Ther..

[174] Pak F., Takavar A., Nedaie H., Rad H.S., Vaezzadeh V., Eqlimi E., Moghadam M.S. (2016). Basic investigation on performance of low-density polymer gel dosimeter. Int. J. Radiat. Res..

[175] Bloembergen N., Purcell E.M., Pound R.V. (1948). Relaxation effects in nuclear magnetic resonance absorption. Phys. Rev..

[176] Dewhurst H. (1951). Effect of Organic Substances on the *γ*-Ray Oxidation of Ferrous Sulfate. J. Chem. Phys..

[177] Collura G., Gallo S., Tranchina L., Abbate B.F., Bartolotta A., d’Errico F., Marrale M. (2018). Analysis of the response of PVA-GTA Fricke-gel dosimeters with clinical magnetic resonance imaging. Nucl. Instrum. Methods Phys. Res. Sect. B Beam Interact. Mater. Atoms.

[178] Marrale M., Brai M., Gagliardo C., Gallo S., Longo A., Tranchina L., Abbate B., Collura G., Gallias K., Caputo V. (2014). Correlation between ferrous ammonium sulfate concentration, sensitivity and stability of Fricke gel dosimeters exposed to clinical X-ray beams. Nucl. Instrum. Methods Phys. Res. Sect. B Beam Interact. Mater. Atoms.

[179] Marrale M., Brai M., Longo A., Gallo S., Tomarchio E., Tranchina L., Gagliardo C., d’Errico F. (2014). NMR relaxometry measurements of Fricke gel dosimeters exposed to neutrons. Radiat. Phys. Chem..

[180] Gallo S., Collura G., Iacoviello G., Longo A., Tranchina L., Bartolotta A., d’Errico F., Marrale M. (2017). Preliminary magnetic resonance relaxometric analysis of Fricke gel dosimeters produced with polyvinyl alcohol and glutaraldehyde. Nucl. Technol. Radiat. Prot..

[181] Schreiner L. (2004). Review of Fricke gel dosimeters. J. Phys. Conf. Ser..

[182] Maryanski M., Zastavker Y., Gore J. (1996). Radiation dose distributions in three dimensions from tomographic optical density scanning of polymer gels: II. Optical properties of the BANG polymer gel. Phys. Med. Biol..

[183] Keshtkar M., Takavar A., Zahmatkesh M., Montazerabadi A. (2017). Uncertainty analysis in MRI-based polymer gel dosimetry. J. Biomed. Phys. Eng..

[184] De Deene Y., De Wagter C., Van Duyse B., Derycke S., De Neve W., Achten E. (1998). Three-dimensional dosimetry using polymer gel and magnetic resonance imaging applied to the verification of conformal radiation therapy in head-and-neck cancer. Radiother. Oncol..

[185] Lepage M., McMahon K., Galloway G., De Deene Y., Bäck S.Å.J., Baldock C. (2002). Magnetization transfer imaging for polymer gel dosimetry. Phys. Med. Biol..

[186] Skorupa A., Woźnica A., Ciszek M., Staniszewski M., Kijonka M., Kozicki M., Woźniak B., Orlef A., Polański A., Boguszewicz Ł (2020). Application of high field magnetic resonance microimaging in polymer gel dosimetry. Med. Phys..

[187] Quevedo A., Luo G., Galhardo E., Price M., Nicolucci P., Gore J.C., Zu Z. (2018). Polymer gel dosimetry by nuclear Overhauser enhancement (NOE) magnetic resonance imaging. Phys. Med. Biol..

[188] Doran S.J. (2009). The history and principles of chemical dosimetry for 3-D radiation fields: Gels, polymers and plastics. Appl. Radiat. Isot..

[189] Maryanski M.J., Ranade M.K. (2001). Laser microbeam CT scanning of dosimetry gels. Proceedings of the Medical Imaging 2001: Physics of Medical Imaging, San Diego, CA, USA, 17–22 Ferbruary 2001.

[190] Van Doorn T., Bhat M., Rutten T., Tran T., Costanzo A. (2005). A fast, high spatial resolution optical tomographic scanner for measurement of absorption in gel dosimetry. Australas. Phys. Eng. Sci. Med..

[191] Wuu C.S., Schiff P., Maryanski M.J., Liu T., Borzillary S., Weinberger J. (2003). Dosimetry study of Re-188 liquid balloon for intravascular brachytherapy using polymer gel dosimeters and laser-beam optical CT scanner. Med. Phys..

[192] Massillon-Jl G., Minniti R., Mitch M.G., Maryanski M., Soares C.G. (2009). The use of gel dosimetry to measure the 3D dose distribution of a 90Sr/90Y intravascular brachytherapy seed. Phys. Med. Biol..

[193] Massillon-Jl G., Minniti R., Soares C., Maryanski M., Robertson S. (2010). Characteristics of a new polymer gel for high-dose gradient dosimetry using a micro optical CT scanner. Appl. Radiat. Isot..

[194] Ramm D., Rutten T.P., Shepherd J., Bezak E. (2012). Optical CT scanner for in-air readout of gels for external radiation beam 3D dosimetry. Phys. Med. Biol..

[195] Shih C.T., Chang Y.J., Hsu J.T., Chuang K.S., Chang S.J., Wu J. (2015). Image reconstruction of optical computed tomography by using the algebraic reconstruction technique for dose readouts of polymer gel dosimeters. Phys. Med..

[196] Hilts M., Audet C., Duzenli C. (1999). X-ray computer tomography for polymer gel dosimetry. Med. Phys..

[197] Kakakhel M., Jirasek A., Johnston H., Kairn T., Trapp J. (2017). Improving the quality of reconstructed X-ray CT images of polymer gel dosimeters: Zero-scan coupled with adaptive mean filtering. Australas. Phys. Eng. Sci. Med..

[198] Maynard E., Heath E., Hilts M., Jirasek A. (2018). Introduction of a deformable x-ray CT polymer gel dosimetry system. Phys. Med. Biol..

[199] Javaheri N., Yarahmadi M., Refaei A., Aghamohammadi A. (2020). Improvement of sensitivity of X-ray CT reading method for polymer gel in radiation therapy. Rep. Pract. Oncol. Radiother..

[200] Hayati H., Mesbahi A., Nazarpoor M. (2016). Monte Carlo modeling of a conventional X-ray computed tomography scanner for gel dosimetry purposes. Radiol. Phys. Technol..

[201] Jirasek A., Johnston H., Hilts M. (2015). Dose rate properties of NIPAM-based x-ray CT polymer gel dosimeters. Phys. Med. Biol..

[202] Mather M.L., Baldock C. (2003). Ultrasound tomography imaging of radiation dose distributions in polymer gel dosimeters: Preliminary study. Med. Phys..

[203] Atkins T.J., Humphrey V., Duck F., Tooley M. (2010). Investigation of ultrasonic properties of MAGIC gels for pulse-echo gel dosimetry. J. Phys. Conf. Ser..

[204] Khoei S., Trapp J.V., Langton C.M. (2014). Ultrasound attenuation computed tomography assessment of PAGAT gel dose. Phys. Med. Biol..

[205] Siti K., Iskandar S., Azhar A., Ramzun M., Halimah M.K. (2014). Acoustic evaluation of hema polymer gel dosimeter phantoms. Adv. Mater. Res..

[206] Masoumi H., Mokhtari-Dizaji M., Arbabi A., Bakhshandeh M. (2016). Determine the dose distribution using ultrasound parameters in MAGIC-f polymer gels. Dose-Response.

[207] Goharpey N., Mokhtari-Dizaji M., Bakhshandeh M. (2020). A Novel Ultrasonic Gel Phantom Dosimetry for Evaluation of the Dose Response. J. Korean Phys. Soc..

[208] Crescenti R.A., Bamber J.C., Oberai A.A., Barbone P.E., Richter J.P., Rivas C., Bush N.L., Webb S. (2010). Quantitative ultrasonic elastography for gel dosimetry. Ultrasound Med. Biol..

[209] Vieira S.L., de Oliveira L.N., Carneiro A.A.O. (2019). Quantitative magnetic resonance elastography for polymer-gel dosimetry phantoms. Med. Eng. Phys..

[210] Xie W.H., Su C.T., Kao Y.C.J., Chang T.H., Chang Y.J., Yao C.H., Hsieh B.Y. (2020). Radiotherapy dose characterization of gel dosimetry using shear wave elasticity imaging. Med Phys..

